# An ecotype-specific effect of osmopriming and melatonin during salt stress in *Arabidopsis thaliana*

**DOI:** 10.1186/s12870-024-05434-5

**Published:** 2024-07-25

**Authors:** Michał Juraniec, Erik Goormaghtigh, Małgorzata M. Posmyk, Nathalie Verbruggen

**Affiliations:** 1 Department of Plant Ecophysiology, Faculty of Biology and Environmental Protection, University of Lodz, Lodz, 90 237 Poland; 2 Laboratory for the Structure and Function of Biological Membranes, Center for Structural Biology and Bioinformatics, Faculté des Sciences, Université libre de Bruxelles, Brussels, 1050 Belgium; 3 Laboratoire de Physiologie et de Génétique Moléculaire des Plantes, Faculté des Sciences, Université libre de Bruxelles, Brussels, 1050 Belgium

**Keywords:** *Arabidopsis thaliana*, Ecotypes, Salt stress, Seed priming, Root architecture, Melatonin, Stress markers, Antioxidant enzymes, Oxidative stress

## Abstract

**Background:**

Natural populations of *Arabidopsis thaliana* exhibit phenotypic variations in specific environments and growth conditions. However, this variation has not been explored after seed osmopriming treatments. The natural variation in biomass production and root system architecture (RSA) was investigated across the *Arabidopsis thaliana* core collection in response to the pre-sawing seed treatments by osmopriming, with and without melatonin (Mel). The goal was to identify and characterize physiologically contrasting ecotypes.

**Results:**

Variability in RSA parameters in response to PEG-6000 seed osmopriming with and without Mel was observed across *Arabidopsis thaliana* ecotypes with especially positive impact of Mel addition under both control and 100 mM NaCl stress conditions. Two ecotypes, Can-0 and Kn-0, exhibited contrasted root phenotypes: seed osmopriming with and without Mel reduced the root growth of Can-0 plants while enhancing it in Kn-0 ones under both control and salt stress conditions. To understand the stress responses in these two ecotypes, main stress markers as well as physiological analyses were assessed in shoots and roots. Although the effect of Mel addition was evident in both ecotypes, its protective effect was more pronounced in Kn-0. Antioxidant enzymes were induced by osmopriming with Mel in both ecotypes, but Kn-0 was characterized by a higher responsiveness, especially in the activities of peroxidases in roots. Kn-0 plants experienced lower oxidative stress, and salt-induced ROS accumulation was reduced by osmopriming with Mel. In contrast, Can-0 exhibited lower enzyme activities but the accumulation of proline in its organs was particularly high. In both ecotypes, a greater response of antioxidant enzymes and proline accumulation was observed compared to mechanisms involving the reduction of Na^+^ content and prevention of K^+^ efflux.

**Conclusions:**

In contrast to Can-0, Kn-0 plants grown from seeds osmoprimed with and without Mel displayed a lower root sensitivity to NaCl-induced oxidative stress. The opposite root growth patterns, enhanced by osmopriming treatments might result from different protective mechanisms employed by these two ecotypes which in turn result from adaptive strategies proper to specific habitats from which Can-0 and Kn-0 originate. The isolation of contrasting phenotypes paves the way for the identification of genetic factors affecting osmopriming efficiency.

**Supplementary Information:**

The online version contains supplementary material available at 10.1186/s12870-024-05434-5.

## Background

Soil salinity causes both osmotic stress and ionic toxicity and is one of the most limiting and detrimental factors to crop production worldwide [1, 2]. Salinity delays, or even inhibits, seed germination and affects seedling development [3–5]. Salinized plants suffer from dehydration and lower nutrient uptake which generates metabolic toxicity, nutritional stress, membrane dysfunction and oxidative stress [1, 6]. All these physiological disturbances lead to tissue damage, reduction in organ growth and biomass production as well as early senescence. Although shoot growth is more sensitive to salt stress than root growth [7], root is the first organ to encounter and respond to soil salinity. The response of the root system architecture (RSA) to the soil environment and modifications of its (three-dimensional) structure is one of the best examples of plant developmental plasticity. This trait is essential for the ability to forage soil for water and mineral nutrients and determines acclimation of plants to multiple stresses [8]. RSA is shaped by the pattern of root branching – formation of lateral roots (LRs) and by the rate and direction of growth of individual roots [9]. As observed in various species, RSA is significantly modified in response to high NaCl concentrations by repressing the formation of LRs and reducing elongation of both primary roots (PRs) and LRs [10–14].

The pre-exposure to a mild stress, a so called priming or conditioning, at early developmental stage elicits a stress memory (or priming memory), which renders plants more responsive to secondary exposure to stresses and thus represents an attractive strategy to improve crop resilience [15, 16]. Stress priming generates changes at the molecular levels, involving epigenetic modifications in DNA, induces the expression of favorable traits and enables plants to be more tolerant to additional severe stresses [17–22]. Stress memory can be established by both plant and seed priming, and confers a so-called cross-stress tolerance [23, 24]. Seed priming involves controlled seed hydration to initiate germination-related biophysical and biochemical processes while preventing the radicle from emergence, and is followed by seed re-drying to their initial water content [25]. Priming enables the transition of the seed from a quiescent to a germinating state involving the initiation of germination-related activities e.g. respiration, endosperm weakening, hydrolysis of abscisic acid, gene expression. It also imposes abiotic stress, known as stress imprinting characterized by the repression of radicle protrusion and stimulation of stress-responsive elements including the activation of antioxidative systems (both antioxidant enzymes and small-molecule antioxidants) and the accumulation of LEA proteins [23]. Thus, in contrast to plant priming, seed priming, by encompassing two components: advanced germination status and initial stress imprint, confers greater stress tolerance. Seed germination and emergence, a critical phase of plant development, is very sensitive to abiotic stresses and may lead to retarded growth and lower yield if it occurs under unfavorable environmental conditions. Seed priming was developed as a cost-effective and rapid method improving the germination parameters, faster and more uniform germination and emergence, which have practical implications to overcome the negative effects of adverse environmental conditions [26–28]. Moreover, seed priming was also shown to improve seedling establishment, plant growth, yield and tolerance against disadvantageous conditions, including drought and salinity stress, in numerous plant species [29–33]. Seed priming can be combined with the application of growth regulators or bioactive substances – biostimulators that may additionally improve defense mechanisms and confer higher stress tolerance as it was found in many crops and non-crop species [34–36]. The most commonly used biostimulators are hormones (auxin, gibberellin), mineral nutrients (magnesium, zinc), vitamins (ascorbate), osmoregulators (mannitol, proline, trehalose), polyamines, melatonin, sodium hydrosulfide or hydrogen sulfide. Moreover, several studies have also showed that pre-sawing seed priming can improve growth and volume of root system and shoot biomass. Kaur et al. [37] reported that chickpea plants obtained from seeds primed with mannitol developed longer roots and shoots with higher fresh weights under drought conditions than plants obtained from non-primed seeds. Similarly, rice plants obtained from seeds primed with proline developed root systems with enlarged volume, length and degree of branching [38].

Exogenously applied during plant growth or seed priming, melatonin (Mel) has been found to be an excellent biostimulator, conferring resistance to several biotic and abiotic stresses. It was shown to protect both monocotyledonous and dicotyledonous plant species against various stresses or to alleviate their consequences including drought [39–42], salinity [43–47], cold [48–50], high temperatures [51], flooding [52], low phosphorus tolerance [53] or trace metal excess [54–56]. Mel is a highly conserved indoleamine present in evolutionary distant organisms, from bacteria and algae to vertebrates and higher plants. Although in plants, Mel was shown to exhibit pleiotropic activities including the involvement in growth and developmental processes, shoot and root morphogenesis control, retarded leaf senescence and protection of developing tissues [57, 58], the primary function of Mel is its superior antioxidant capacity as it functions as a direct radical scavenger, regulator of antioxidant enzymes to detoxify ROS indirectly as well as glutathione synthesis stimulator [59–61]. A growing body of evidence shows that Mel participates in plant acclimatory reactions to environmental stresses by modifying metabolism, improving photosynthetic capacity and activating various antioxidant responses [31, 40–43, 46, 52, 62–66]. Interestingly, in several species exogenously applied Mel has been also found to modulate root growth by promoting PR elongation and enhancing root branching – lateral and adventitious root formation [67, 68]. Its action was especially beneficial for plants subjected to drought or NaCl stress [39, 69, 70]. Mel was shown to regulate RSA by activating auxin signaling and modulating auxin distribution in roots [71, 72].

In the present work, we analyzed the variability of the effect of seed osmopriming with and without Mel on biomass and main RSA parameters in an *Arabidopsis thaliana* core collection grown under 100 mM NaCl stress conditions. The responses to salt stress of two selected contrasting ecotypes, grown from osmoprimed or non-primed seeds, were further studied in detail.

## Materials and methods

### Plant material

*Arabidopsis thaliana* core collection was obtained from the Genomic Resource Center INRA-Versailles (France) and consisted of 23 ecotypes: Akita, Alcalá de Hernares (Alc-0), Bologna-1 (Bl-1), Bulhary-1 (Blh-1), Burren-0 (Bur-0), Canary Island-0 (Can- 0), Catania-1 (Ct-1), Cape Verde Islands-0 (Cvi-0), Edinburgh-0 (Edi-0), Geneva-0 (Ge-0), Greenville-0 (Gre-0), St Jean Cap Ferrat (Jea), Kaunas-0 (Kn-0), Mulhen-1 (Mh-1), Martuba-0 (Mt-0), Konchezero (N13), Oystese-0 (Oy-0), Pyla-1 (Pyl-1), Sakata, Shahdara (Sha), Stockholm-0 (St-0), Stobowa-0 (Stw- 0), and Tsushima (Tsu-0). Ibel Tazekka-0 (Ita-0) ecotype was discarded from the study due to poor germination rate, flowering and insufficient number of amplified seeds for carrying experiments. The collection was completed by the reference Columbia-0 (Col-0) ecotype and by six additional accessions, including Ameland-1(Amel-1), De Waal-2 (Dew-2), De Horse-2 (Dhs-2), Lessine-1 (Less-1), Llagostera-0 (Ll-0) and Noordwijk-1 (Nok-1), characterized by contrasted root morphology and collected in Belgium, Holland and Spain. Seeds of the same age were used for experiments (batches of seeds were simultaneously generated under the greenhouse conditions from the original stocks).

### Priming treatments and melatonin application

Plants used for all experiments described herein were grown from three variants of seeds: the osmoprimed seeds (O), the seeds osmoprimed with melatonin (OMel) and the non-treated control seeds (NT). O seeds were prepared as described [73] by incubating mature seeds at 20 °C in -0.75 MPa PEG-6000 water solution, during 7 days in the darkness. OMel seeds were prepared similarly as O seeds, but − 0.75 MPa PEG-6000 was prepared in this case with water solution of 50 µM Mel (Sigma Aldrich, catalog number: M5250). Subsequently, both seed variants, O and OMel, were quickly washed with distilled water to remove PEG and dried to initial water content for few days in circulating air at room temperature. The concentration of Mel applied into the OMel seeds was selected experimentally on the basis of the preliminary measurements of selected RSA parameters in Col-0 ecotype.

### Melatonin extraction and HPLC-MS/MS quantification

50 mg of dry seeds were grinded to a fine powder in pre-cooled glass tubes with the addition of absolute ethanol using an electric homogenizer. The homogenates were incubated for three hours in a cold room in darkness with constant shaking to ensure complete extraction of Mel and were subsequently centrifuged at 20 000 x *g* for 10 min at 4 °C. The supernatant was filtered (ISO-Disc filters PTEF-4-2 4 mm x 0.2 μm) and diluted with distilled water for the analysis. Mel was quantified by high-performance liquid chromatography as described [74] using an Agilent 1200 LC System coupled with AB Sciex 4500 QTRAP mass detector equipped with TurboSpray Ion Source. Analysis was performed with three technical replicates for each of three portions of seeds of each ecotype and each seed treatment variant. The working range for the quantitation covered the linearity of the standard curves from 0.1 to 100 ng ml^− 1^.

### Growth conditions and stress treatment

Seeds were surface sterilized with ethanol 70% (v/v) during 5 min, with a 20% (v/v) HClO solution during 5 min and rinsed with sterile water. Seeds were subsequently sown on agar plates containing half-strength Murashige and Skoog (MS) medium including vitamins (Duchefa, Haarlem, the Netherlands), 10 g l^− 1^ sucrose, 0.5 g l^− 1^ MES and 8 g l^− 1^ plant agar. After 48 h of stratification at 4 °C, seedlings were grown vertically for 7 days in a culture chamber at 21 °C and under a daylight regime of 16 h (100 µmol photons m^− 2^ s^− 1^) and 8 h darkness. After that period, seedlings were transferred onto the same medium supplemented with or without 100 mM NaCl and allowed to grow for next 7 days. These growing conditions were used for all experiments described herein. Additionally, H_2_O_2_ accumulation, antioxidant enzyme activities and ethylene emanation were also analyzed in seedlings grown in the presence of 100 mM NaCl for 24 h to evaluate short-time NaCl stress treatment.

### Root system architecture analysis

14-d-old plants were used for the RSA analysis. Three plates containing five seedlings per ecotype (30 genotypes), per condition (control and salt stress) and per seed treatment variant (NT, O and OMel) were measured (*n* = 15). Lateral roots were disentangled on the plates for facilitating image analysis. Roots were scanned with a Perfection V30 (Seiko Epson, Tokyo) scanner at 400 dpi and images were analyzed with RootNav software [75]. Trait measurement computations were performed with RootNav Viewer. Specific trait measurements were programmed in C# and included in RootNav Viewer as a plugin. To avoid artefacts, only lateral roots > 1 mm long were considered for morphological trait calculations. Parameters characterizing RSA are listed and detailed in Fig. S1. Collected RSA parameters confirmed the previously observed phenotype of candidate ecotypes grown under control and salt stress conditions. Following analysis of RSA, roots and shoots of five plants from each of three plates were pooled to measure total fresh weight.

### Mineral analysis

Shoots and roots of 2-week-old plants were harvested, rinsed with deionized water for 1 min, dried at 65 °C for 48 h and crushed. Weighed material was digested with 7 M nitric acid for 6 h at 130 °C. Samples were assayed by the double-beam atomic absorption spectrometer (Perkin-Elmer model 3110, USA). Quantification of sodium and potassium was performed with three technical replicates for each of three biological repeats. The data were expressed as mg g^− 1^ of DW.

### TBARS test

The levels of lipid peroxidation were assessed by the quantification of their peroxidation products. Thiobarbituric acid reactive substances (TBARS): aldehydes and carbonyl-containing compounds were determined as described [76].

### Proline quantification

150 mg of plant material was ground with 3% sulfosalicylic acid and proline concentration in organs was determined according to Bates et al. [77]. Quantification was performed with three technical replicates for each of three biological repeats. The data were expressed as mg of proline per 1 g of FW.

### Hydrogen peroxide quantification

Measurement of H_2_O_2_ was performed with the Amplex™ Red hydrogen peroxide/peroxidase assay kit (Invitrogen by Thermo Fisher Scientific, Life Technologies, USA, catalog number: A22188) according to the manufacturer’s instructions. In brief, roots and shoots were grinded in liquid nitrogen, weighed and resuspended in an appropriate volume of ice-cold phosphate buffer. Samples were centrifuged for 3 min at 16 200 x *g* at 4 °C and the obtained supernatant was used immediately with H_2_O_2_ measurements. Individual components of the reaction mix were added to each well of the plate following the order provided by the manufacturer. For each plate, three negative controls were run: sample background (without Amplex™ Red reagent), negative control without H_2_O_2_ and Amplex™ Red background (without H_2_O_2_ and horseradish peroxidase enzyme). The reactions were incubated at room temperature for 30 min in the dark and read using a plate reader at 560 nm (reference wavelength 650 nm) with two technical replicates for each of two biological repeats. The concentrations were calculated using a standard curve ranging from 0 to 20 µM H_2_O_2_.

### Antioxidant enzyme activity assays

FW shoots or roots were homogenized on ice with ~ 2% PVP in 0.1 M phosphate buffer (pH 7.5) supplemented with 2.5 mM DTT, 1 mM EDTA, 1.25 mM PEG-4000 and 1 mM PMSF protease inhibitor and centrifuged at 16 000 x *g* for 30 min at 4 °C. The supernatant was filtered through Miracloth, desalted on PD-10 column (GE Healthcare, Sweden) and used for assays. The protein content in extracts was determined according to Bradford method [78]. The enzymes, including superoxide dismutase (SOD) EC 1.15.1.1, catalase (CAT) EC 1.11.1.6, ascorbate peroxidase (APX) EC 1.11.1.11, guaiacol peroxidase (POX) EC 1.11.1.7, glutathione peroxidase (GSH-PX) EC 1.11.1.9, glutathione reductase (GSSG-R) EC 1.6.4.2 were assayed as described [79]. Enzyme activities were measured with three technical replicates for each of three biological repeats for each condition and seed variant. SOD activity was expressed as the enzyme unit per 1 mg of protein [U mg_PROTEIN_^−1^] whereas the activities of other enzymes were expressed as micromoles of decomposed or oxidized substrate during 1 min per 1 mg of proteins [µmol min^− 1^ mg_PROTEIN_^−1^].

### Ethylene measurements

13-d-old plants grown under control conditions were transferred onto the same or 100 mM NaCl-containing MS medium for 24 h prior to ethylene production measurements. Headspace samples from both growth conditions were analyzed with the laser-based ETD-300 Sensor-Sense photoacoustic ethylene detector (Nijmegen, the Netherlands). The bottom part of the plate with the agar was covered with a glass plate with an inlet and outlet for gas flow. The system was tied together with two metal pieces. Ethylene production in six plates placed vertically in a growth chamber was measured sequentially with an automated gas sampling performed under a stop-and-flow schedule. Gas accumulated in each plate during 50 min was flushed to the detector during 10 min at a flow rate of 3 l h^− 1^. The ethylene accumulation in six plates was then measured alternately over at least 12 h during the day period and the average ethylene emanation was calculated. The experiment was run in three biological replicates for each ecotype, seed variant and growth conditions.

### Statistical analysis

The analysis of variance (ANOVA) with *p*-value < 0.05 set as the cut-off value, as indicative of significant differences between the means, followed by a Duncan *post-hoc* test was performed using the Statistica software (StatSoft, Inc). ANOVA results are provided in supplementary materials. The hierarchical cluster analysis was performed with Kinetics, a custom-made program, running under Matlab 7.1 (Matlab, Mathworks Inc.). The classification procedure is based on the discriminant variables selection and enables a hierarchical clustering of groups with minimum loss of information [80]. The cluster method is based on the similarity among group members with respect to many variables. In order to evidence the RSA variations induced by salt stress, the mean values of analyzed RSA parameters of control plants were subtracted from the mean values of salt-stressed plants in corresponding ecotypes. The differences obtained for each ecotype represent the actual modifications in RSA caused by salt stress. These discriminant values were used to calculate the Euclidean distance between the ecotype pairs and to construct a cluster dendrogram based on Ward’s algorithm. The parameters used in this analysis were: the total fresh plant biomass, L_PR_, N_LR_ and ΣL_LR_. Three dendrograms corresponding to the three seed variants were calculated.

## Results

### Variability of biomass production and root architectures in response to seed priming

**Fig. 1:**
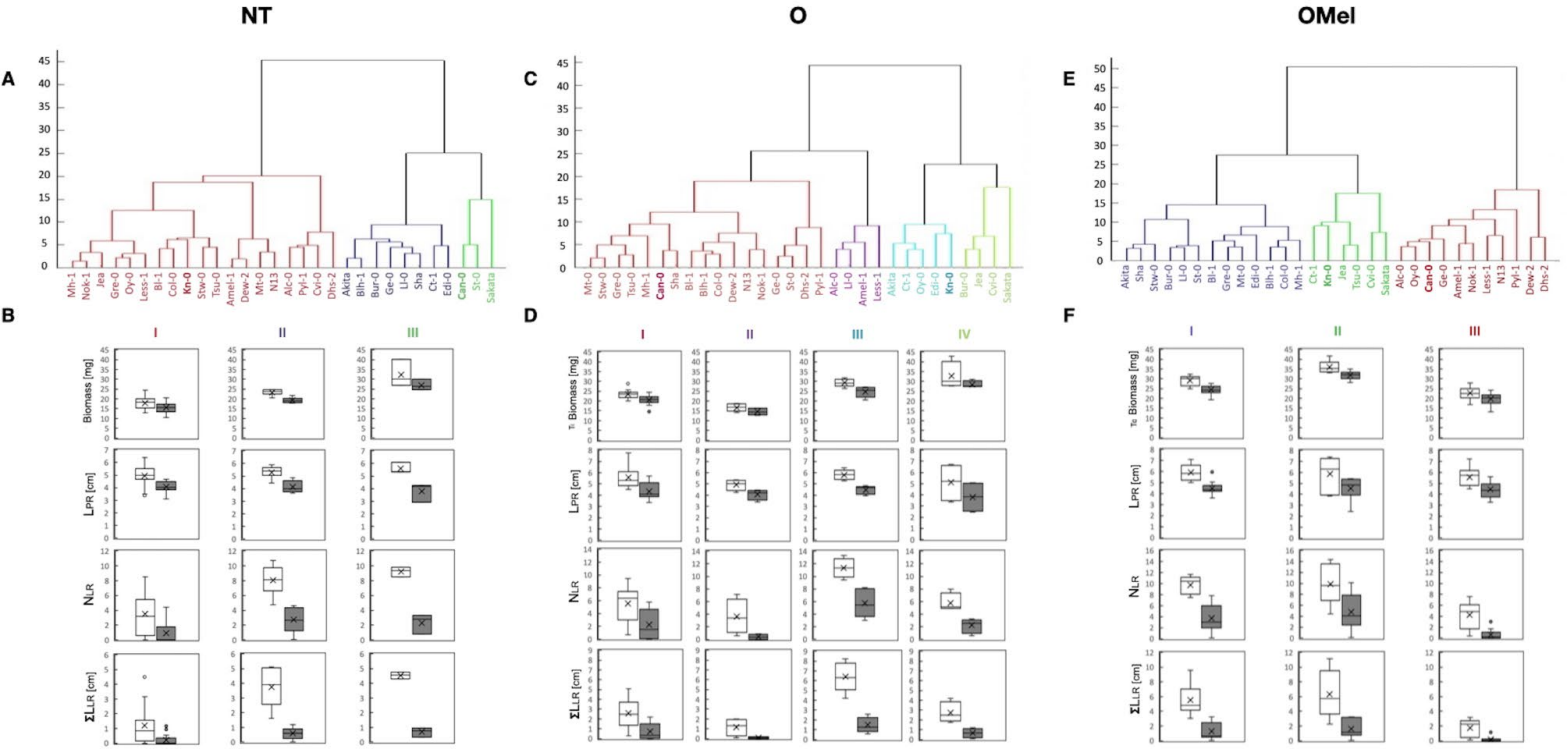
Ascending hierarchical classifications of *Arabidopsis thaliana* ecotypes grown in vitro from three seed variants. 30 ecotypes, grown from non-treated (NT) seeds (**A**), osmoprimed (O) seeds (**C**) and seeds osmoprimed with melatonin (OMel) (**E**) are divided into distinct groups (I, II, III or IV) on the basis of cluster analysis. Box plots show the performance of these groups for biomass production and root architecture under control (white boxes) and 100 mM NaCl stress (grey boxes) conditions: total fresh weight, length of primary root (L_PR_), number of lateral roots (N_LR_) and sum of lateral root length (ΣL_LR_) for plants grown from NT (**B**), O (**D**) and OMel seeds (**F**). The graphs are built using one median value from each ecotype from control or salt stress conditions. The boxes represent the interquartile range and the wiskers extend to the most extreme data point within 1.5 x interquartile range from the box. Crosses indicate the mean values while the outliers are shown as circle points. For the sake of the statistical comparison, the control values were not subtracted here. Biomass trait: *n* = 3 observations (3 × 5 pooled plants), root traits: *n* = 15 observations

Mature seeds of thirty *Arabidopsis thaliana* ecotypes were osmoprimed with (OMel) and without Mel (O). One-week-old plants obtained from OMel and O seeds as well as from NT ones were transferred for one week onto MS/2 control medium or the same medium supplemented with 100 mM NaCl to evaluate the variability in RSA across the ecotypes. Total fresh plant biomass and three main RSA parameters i.e. length of primary root (L_PR_), number of lateral roots (N_LR_) and sum of lateral root lengths (ΣL_LR_) (Fig. S1) were analyzed. In the presence of 100 mM NaCl, the biomass (Fig. S2) as well as the three RSA parameters (Fig. S3-5) were reduced in most ecotypes in comparison with the control conditions. Overall seed treatment by osmopriming with and without Mel had a clear and generally positive impact on measured traits in the majority of ecotypes under both conditions and induced a phenotypic variability. The greatest performance was observed in plants grown from seeds osmoprimed with Mel, however, some ecotypes showed reduced or even negative responsiveness towards seed osmopriming. For instance, Can-0 plants grown from O and OMel seeds showed a decrease in biomass production in comparison with NT seeds under both control and stress conditions (Fig. S2). In Can-0 and Sakata, the PRs in OMel variants were shorter than in the NT ones grown under both control and NaCl stress conditions (Fig. S3). Similarly, the N_LR_ and ΣL_LR_ in OMel variants of these two ecotypes were also lower than these in the NT ones (Fig. S4 and S5). To discriminate RSA differences and find the most contrasting ecotypes, the three main RSA traits and the total biomass measured in plants grown under salt stress conditions were compared with those of the unstressed plants and an ascending hierarchical classification using Ward’s algorithm was performed on the basis of calculated differences. Distinct groups of ecotypes with most similar RSA parameters were identified for plants obtained from each of the three treatment variants. Three distinct groups emerged from the analysis among NT plants, four among plants grown from seeds osmoprimed without Mel and three groups among plants obtained from seeds osmoprimed with Mel (Fig. 1A, C and E). A statistical comparison of the groups, reported as boxplots in Fig. 1, was performed to identify common characteristics shared by the ecotypes belonging to distinct groups. In the NT plants, ecotypes belonging to group III (Can-0, St-0 and Sakata) were characterized by higher biomass under both control and stress conditions than ecotypes of the group I and II (Fig. 1B). Ecotypes from groups II (Akita, Blh-1, Bur-0, Ge-0, Ll-0, Sha, Ct-1 and Edi-0) and III had generally more LRs and showed higher values of ΣL_LR_ than ecotypes from group I. Finally, the ecotypes from group III displayed the biggest differences in L_PR_, N_LR_ and ΣL_LR_ between control and stress conditions. Among ecotypes grown from O seeds, groups III (Akita, Ct-1, Oy-0, Edi-0 and Kn-0) and IV (Bur-0, Jea, Cvi-0 and Sakata) were characterized by the highest biomass while group III had higher N_LR_ and ΣL_LR_ than other groups under both culture conditions (Fig. 1D). In the plants grown form OMel seeds, group II (Ct-1, Kn-0, Jea, Tsu-0, Cvi-0 and Sakata) had higher biomass as well as higher N_LR_ and ΣL_LR_ than other two groups, especially than group III (Alc-0, Oy-0, Can-0, Ge-0, Amel-1, Nok-1, Less-1, N13, Pyl-1, Dew-2 and Dhs-2) under both conditions (Fig. 1F).

**Fig. 2:**
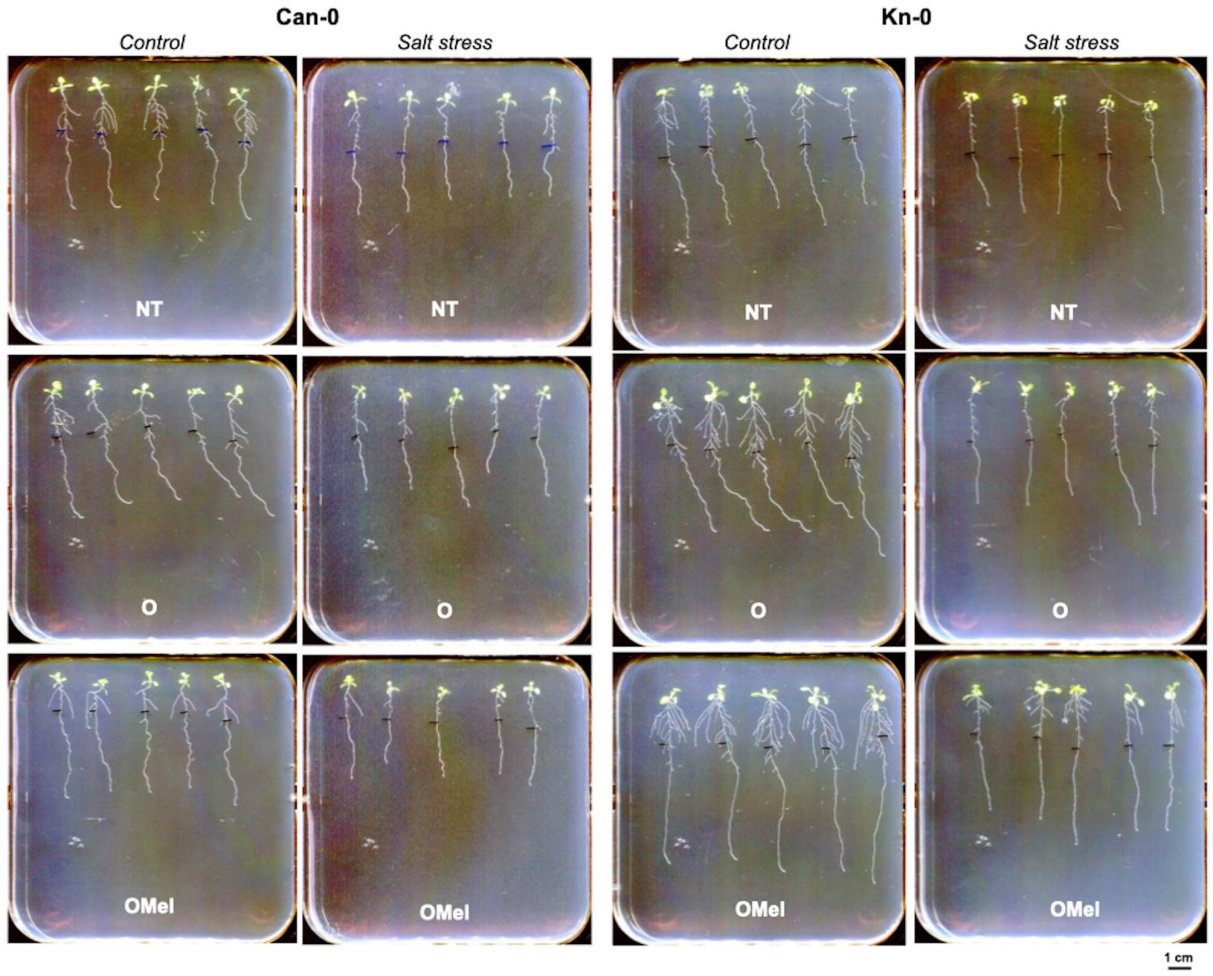
Representative root system architecture of Can-0 and Kn-0 ecotypes in response to salt stress. Seedlings were grown from non-treated (NT), osmoprimed (O) and osmoprimed with melatonin (OMel) seeds on vertical plates on control MS/2 medium and transferred 5 days after germination onto the same medium supplemented with or without 100 mM NaCl. The photographs were taken 7 days after transfer. Bar, 1 cm

**Fig. 3:**
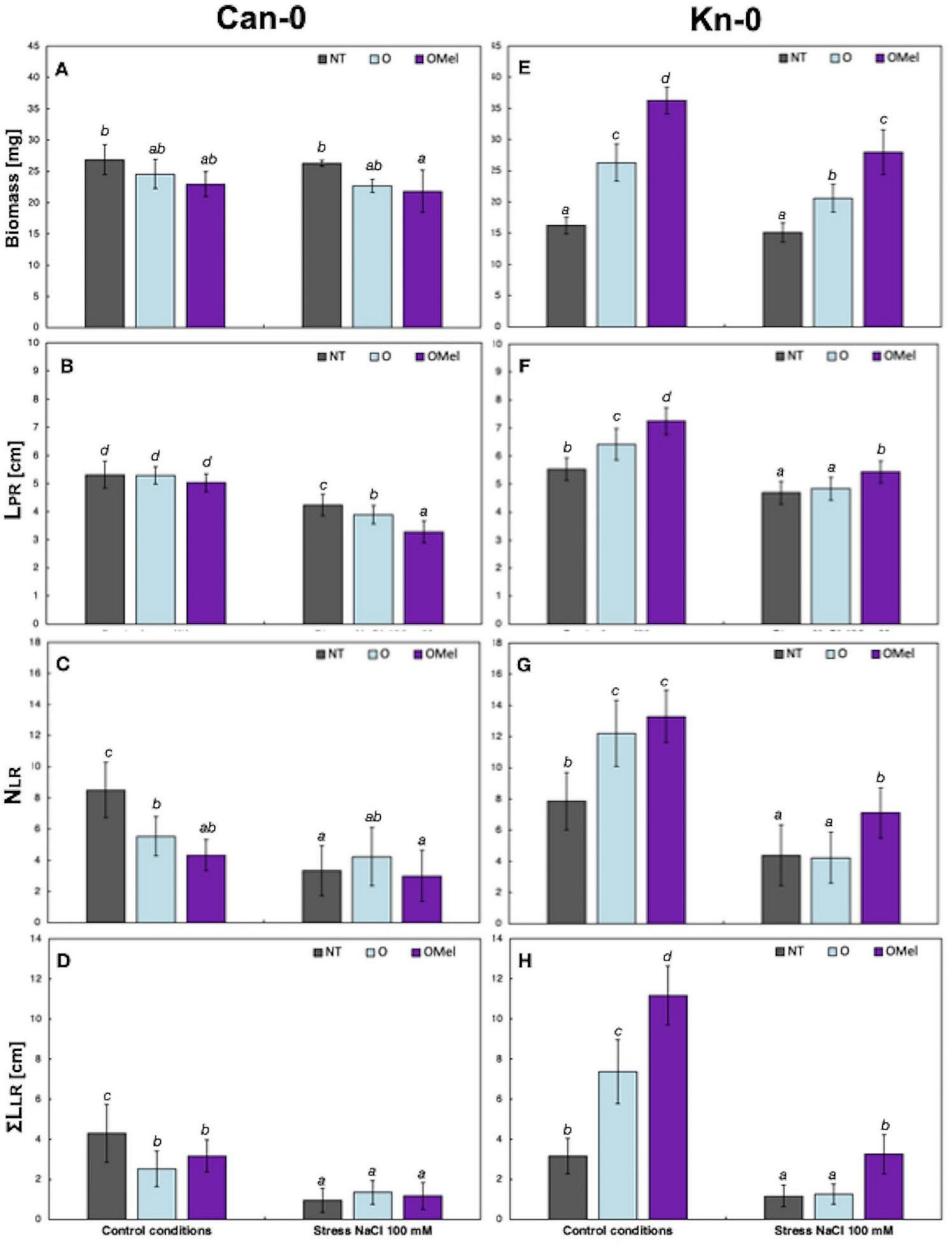
Biomass and principal root architecture parameters measured in in vitro-grown Can-0 and Kn-0 ecotypes. Seedlings were grown from non-treated (NT), osmoprimed (O) and osmoprimed with melatonin (OMel) seeds on vertical plates on control MS/2 medium and transferred 5 days after germination onto the same medium supplemented with or without 100 mM NaCl. Measurements of total fresh weight (**A**, **E**), L_PR_ (**B**, **F**), N_LR_ (**C**, **G**) and ΣL_LR_ (**D**, **H**) were taken 7 days after transfer. Data represent mean values of 3 biological repeats (*n* = 10–15) ± SD. Values representing statistically significant differences at the 5% level (Duncan’s *post-hoc* test) are marked with lowercase letters

**Fig. 4:**
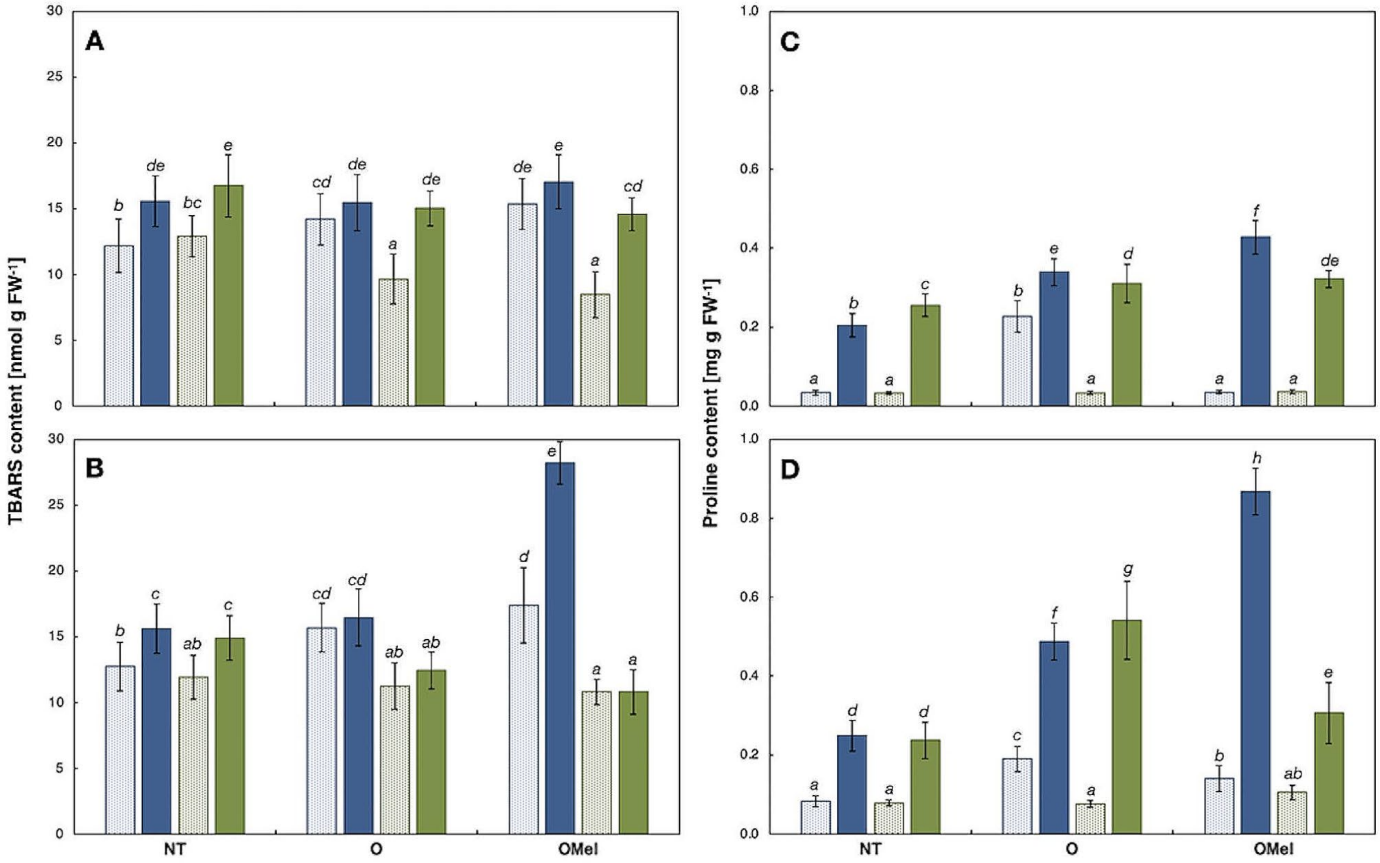
TBARS and proline content in in vitro-grown *Arabidopsis thaliana* Can-0 (blue) and Kn-0 (green) organs. Two-week-old roots (**A**, **C**) and shoots (**B**, **D**), grown from non-treated (NT), osmoprimed (O) and osmoprimed with melatonin (OMel) seeds, were harvested separately one week after plant transfer onto MS/2 medium (control, light bars) or the same medium supplemented with 100 mM NaCl (stress, coloured bars). Data represent mean values of three biological repeats (*n* = 9–15) ± SD. Values representing statistically significant differences at the 5% level (Duncan’s *post-hoc* test) are marked with lowercase letters

**Fig. 5:**
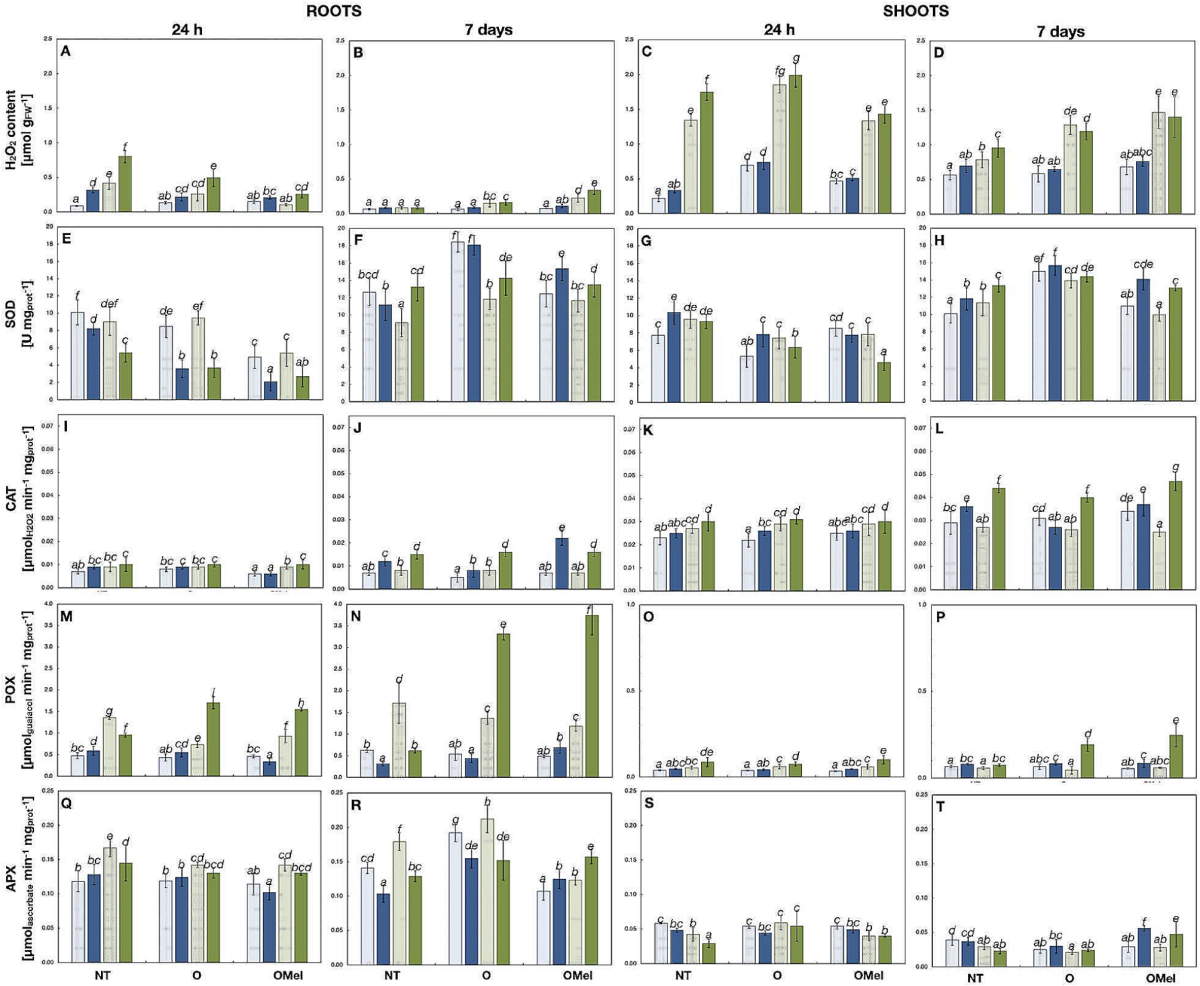
H_2_O_2_ content and antioxidant enzymes activities in *Arabidopsis thaliana* Can-0 (blue) and Kn-0 (green) organs. The activities of superoxide dismutase (SOD), catalase (CAT), guaiacol peroxidase (POX), ascorbate peroxidase (APX) were assayed in roots (**A**, **B**, **E**, **F**, **I**, **J**, **M**, **N**, **Q**, **R**) and shoots (**C**, **D**, **G**, **H**, **K**, **L**, **O**, **P**, **S**, **T**) of one-week-old plants, grown from non-treated (NT), osmoprimed (O) and osmoprimed with melatonin (OMel) seeds, harvested separately 24 h (**A**, **C**, **E**, **G**, **I**, **K**, **M**, **O**, **Q**, **S**) or 7 days (**B**, **D**, **F**, **H**, **J**, **L**, **N**, **P**, **R**, **T**) after transfer onto MS/2 medium (control, light bars) or the same medium supplemented with 100 mM NaCl (stress, coloured bars). Data represent mean values of two biological repeats (*n* = 4) ± SD for H_2_O_2_ content and three biological repeats (*n* = 6–9) ± SD for antioxidant enzyme activities. Values representing statistically significant differences at the 5% level (Duncan’s *post-hoc* test) are marked with lowercase letters

We chose Kn-0 ecotype as one of the most responsive to seed osmopriming with and without Mel and performing the best under both control and salt stress conditions. The least responsive ecotypes were Can-0 and Sakata. Given the small amount of seeds produced by Sakata and the requirement of considerable FW biomass material for physiological tests, we chose Can-0 for further analyses. Both, Kn-0 and Can-0 ecotypes were always clustered in distinct groups in three dendrograms. Representative in vitro phenotypes of Can-0 and Kn-0 plants obtained from three seed variants grown under control and salt stress conditions and collected during the screening experiment are shown on Fig. 2, respectively. The comparison of representative RSA of the NT, O and OMel plant variants of these two ecotypes grown under both culture conditions is shown on Fig. S6. Can-0 ecotype originates from volcanic Canary Islands – Las Palmas/Mirador (Spain) whereas Kn-0 comes from Kaunas (Lithuania). The values of total fresh biomass and three principal RSA parameters of Can-0 and Kn-0 plant variants grown under control and 100 mM NaCl stress are shown on Fig. 3, respectively. These measurements demonstrate the negative effect of seed osmopriming treatments on Can-0 growth and the positive one on Kn-0 growth, especially in Kn-0 plants grown from seeds osmoprimed with the addition of Mel. To better understand the stress responses and the contrasted RSA of two selected ecotypes, physiological analyses were assessed in shoots and roots including hydrogen peroxide and proline production, TBARS, measurements of the activity of antioxidant enzymes, ethylene emanation and mineral profile.

### Mineral profile of Can-0 and Kn-0

To investigate the different response to salt stress of Can-0 and Kn-0, the accumulation of Na^+^ ions and the maintenance of K^+^ concentrations were measured in roots and shoots after one week of growth under stress conditions.

The growth on 100 mM NaCl resulted in a strong increase in sodium content in both organs grown from all types of seeds, but the accumulation was around four times higher in shoots than in roots of both ecotypes (Table 1). In Can-0, O and OMel shoots accumulated approximately 20% less sodium ions than the ones of NT. In Kn-0, the sodium content among all variants was comparable. Can-0 NT and OMel roots accumulated a similar amount of sodium while O roots accumulated approximately 30% more. In Kn-0, both O and OMel roots accumulated around two times more of sodium than NT roots under stress conditions.

**Table 1:** Sodium and potassium content and their ratio in *Arabidopsis thaliana* Can-0 and Kn-0 ecotypes. Two-week-old roots and shoots, grown from non-treated (NT), osmoprimed (O) and osmoprimed with Mel (OMel) seeds, were harvested separately one week after transfer onto MS/2 medium (control) or the same medium supplemented with 100 mM NaCl. Data represent mean values (expressed as mg g^− 1^ of DW) of three biological repeats (*n* = 9–12) ± SD. The multivariate ANOVA analyses were performed on 6 groups of data separated by horizontal lines in the table. Values representing statistically significant differences at the 5% level (Duncan’s *post-hoc* test) between ecotypes and conditions are marked with lowercase letters

Conditions:	Control						Stress NaCl 100 mM					
Ecotypes:	Can-0			Kn-0			Can-0			Kn-0		
**ROOTS**												
*Sodium*												
NT	**0.89**	± 0.19	*a*	**0.50**	± 0.25	*a*	**19.4**	± 4.8	*c*	**13.3**	± 3.1	*b*
O	**1.23**	± 0.21	*a*	**1.61**	± 0.13	*a*	**29.7**	± 5.3	*e*	**26.8**	± 1.7	*de*
OMel	**1.72**	± 0.05	*a*	**1.41**	± 0.15	*a*	**23.1**	± 1.0	*cd*	**23.1**	± 3.3	*c*
*Potassium*												
NT	**40.0**	± 3.3	*d*	**30.0**	± 2.5	*bc*	**33.3**	± 2.8	*c*	**34.7**	± 1.2	*c*
O	**22.6**	± 2.2	*a*	**32.1**	± 3.0	*c*	**27.3**	± 1.0	*ab*	**26.8**	± 1.9	*ab*
OMel	**24.2**	± 1.4	*a*	**25.7**	± 5.5	*ab*	**25.7**	± 2.4	*ab*	**31.9**	± 2.6	*c*
*Ratio Na/K*												
NT	**0.02**	± 0.006	*a*	**0.02**	± 0.008	*a*	**0.58**	± 0.148	*c*	**0.38**	± 0.079	*b*
O	**0.05**	± 0.014	*a*	**0.05**	± 0.007	*a*	**1.09**	± 0.238	*e*	**1.00**	± 0.101	*de*
OMel	**0.07**	± 0.006	*a*	**0.06**	± 0.019	*a*	**0.90**	± 0.086	*d*	**0.72**	± 0.046	*c*
**SHOOTS**												
*Sodium*												
NT	**1.33**	± 0.17	*a*	**0.89**	± 0.25	*a*	**102.2**	± 9.2	*c*	**77.8**	± 6.3	*b*
O	**1.60**	± 0.20	*a*	**1.81**	± 0.14	*a*	**84.2**	± 5.1	*b*	**82.5**	± 10.5	*b*
OMel	**1.85**	± 0.13	*a*	**1.70**	± 0.03	*a*	**87.4**	± 2.9	*b*	**83.5**	± 7.7	*b*
*Potassium*												
NT	**57.7**	± 2.4	*bc*	**64.0**	± 7.4	*cd*	**21.0**	± 4.1	*a*	**25.7**	± 3.2	*a*
O	**54.7**	± 4.2	*b*	**70.3**	± 7.9	*d*	**17.8**	± 0.2	*a*	**18.3**	± 3.6	*a*
OMel	**58.9**	± 5.4	*bc*	**64.2**	± 3.5	*cd*	**19.5**	± 2.6	*a*	**24.2**	± 3.6	*a*
*Ratio Na/K*												
NT	**0.02**	± 0.003	*a*	**0.01**	± 0.003	*a*	**4.87**	± 0.809	*c*	**3.03**	± 0.188	*b*
O	**0.03**	± 0.002	*a*	**0.03**	± 0.002	*a*	**4.73**	± 0.280	*c*	**4.51**	± 0.482	*c*
OMel	**0.03**	± 0.005	*a*	**0.03**	± 0.001	*a*	**4.48**	± 0.559	*c*	**3.45**	± 0.190	*b*

Bold values in the table represent the values of sodium and potassium content and their ratio

Shoots of plants grown from all types of seeds of both ecotypes exposed to salt stress contained around three times less potassium than unstressed shoots (Table 1). Such strong differences were not detected in roots. In Can-0, roots grown from NT seeds accumulated more potassium than these from O and OMel seeds under both conditions, which indicates the negative effect of seed osmopriming. In contrast to NT roots, O and OMel roots grown under stress conditions contained more potassium than unstressed roots. In the Kn-0 ecotype, all variants contained similar amounts of potassium. However, NT and OMel Kn-0 roots contained more K^+^ under stress than under control conditions (Table 1).

Since both ecotypes showed similar K^+^ concentrations in both organs, similar reduction of K^+^ contents in salt-stressed leaves, as well as high and similar Na^+^ accumulation in leaves, we surmised that K^+^ homeostasis and Na^+^ detoxification were similarly sensitive to salinity stress in both ecotypes. The calculated values of the Na^+^/K^+^ ratios in shoots were however lower in Kn-0 than in Can-0 under salt stress conditions, especially in the OMel variants. A similar trend was also observed in Kn-0 roots. Interestingly, these calculations also showed that the Na^+^/K^+^ ratios in O and OMel variants of salt-stressed Can-0 and Kn-0 roots were higher than in corresponding NT roots.

### Quantification of stress markers

Severe salt stress affects many cellular processes and is accompanied by oxidative stress [81]. Products of lipid peroxidation are indicators of oxidative damage. On the other hand, for the osmotic adjustment plants need to synthesize compatible solutes like proline. Proline is also necessary for ROS detoxification, protection of membrane integrity and protein stabilization [82, 83]. Lipid peroxidation (accumulation of TBARS), accumulation of proline and of H_2_O_2_ as well as the antioxidant enzymes activities were measured in both shoots and roots of the two contrasted ecotypes upon one week of stress. Additionally, the accumulation of H_2_O_2_ and activities of antioxidant enzymes were also assayed after short-time (24 h) stress treatment.

In both ecotypes grown from NT seeds a similar ~ 20% increase in TBARS content was measured in salt stressed roots and shoots in comparison with the unstressed plants (Fig. 4A and B). TBARS accumulation also followed an alike decreasing trend in Kn-0 organs under stress conditions. In opposition to Kn-0, seed osmopriming in Can-0 ecotype, and especially with Mel, had a negative effect against oxidative stress induced by NaCl. The highest level of lipid peroxidation was measured in salt stressed Can-0 plants grown from OMel seeds (28.2 nmol/g FW).

Accumulation of proline was triggered in shoots and roots of studied ecotypes by both salt stress and seed osmopriming treatment, but in Can-0 this accumulation was more important (Fig. 4C and D). In both ecotypes, shoots grown from NT seeds accumulated three times more proline under salinity stress than under control conditions, (Fig. 4D). Proline concentrations in NT roots were even higher than in shoots, with a six-fold increase in Can-0 and eight-fold increase in Kn-0 compared with the unstressed plants (Fig. 4C). Seed treatment had a clear effect on proline accumulation in both ecotypes. In Can-0, osmopriming triggered significant proline accumulation in plants grown without stress, its content was over two-fold higher in shoots and almost seven-fold higher in roots in respect to the NT ones (Fig. 4C and D). The highest level of proline accumulation was measured in shoots of salt stressed Can-0 OMel plants (Fig. 4D) A similar trend was observed in Can-0 roots. In Kn-0 salt stressed plants, over two-fold increase in proline accumulation was measured in O shoots in comparison with NT while in the OMel leaves its content was lower (Fig. 4D).

H_2_O_2_ was quantified at two time points: 24 h and 7 d after transfer on MS/2 medium supplemented with 100 mM NaCl. In general, H_2_O_2_ accumulated more after 24 h of stress than after 7 d, and was also more abundant in shoots compared to roots (Fig. 5A-D). After 24 h of stress, the highest concentrations of H_2_O_2_ in roots were measured in plants grown from NT seeds, and the lowest in plants obtained from OMel seeds (Fig. 5A). The accumulation was larger in Kn-0 than in Can-0. In shoots, the difference between Kn-0 and Can-0 was even larger and (Fig. 5B). In NaCl-treated Kn-0 roots, the H_2_O_2_ accumulation was the lowest when seeds were OMel whereas in shoots it was similar whether or not the plants were treated with salt. In Can-0 shoots, the lowest values were measured when seeds were NT. In this ecotype, higher concentrations of H_2_O_2_ were recorded in shoots grown from osmoprimed seeds, with or without Mel. After 7 days of stress treatment, the H_2_O_2_ concentrations were all low in roots and there was no significant increase after NaCl treatment compared to the control medium, except a small increase in OMel Kn-0 (Fig. 5C). In shoots also, there was no increase in H_2_O_2_ due to NaCl treatment (except in NT Kn-0 shoots), but Kn-0 accumulated more H_2_O_2_ than Can-0, especially in shoots from OMel plants (Fig. 5D). However, the difference between the two ecotypes was lower than after 24 h of stress, mainly due to a lower accumulation in Kn-0.

### Quantification of antioxidant enzyme activities

Antioxidant enzyme activities, including total superoxide dismutase (SOD), catalase (CAT), guaiacol peroxidase (POX), ascorbate peroxidase (APX), glutathione peroxidase (GSH-PX) and glutathione reductase (GSSG-R), were quantified in two independent experiments, 24 h and 7 d after transfer of one-week-old plants on MS/2 medium supplemented with 100 mM NaCl. SOD catalyze the partitioning of the highly toxic superoxide radical into O_2_ and H_2_O_2_. CAT decompose H_2_O_2_ to H_2_O and O_2_. Peroxidases catalyze the oxidation of a wide variety of substrates using H_2_O_2_ as the electron acceptor by reducing it to water [84]. GSSG-R restores the pool of reduced glutathione (GSH). Many significant differences in enzyme activities were observed between short-term and long-term salt stress treatments of the two studied ecotypes, between their organs as well as in response to seed osmopriming.

#### Superoxide dismutase

After short-time NaCl treatment total SOD activity in roots was lower in all variants of both ecotypes under stress than under control conditions (Fig. 5E). There was no significant difference between Can-0 and Kn-0 with the exception of NT Kn-0 roots that showed a lower SOD activity. Interestingly, SOD activities under control and stress conditions were lower in roots grown from seeds osmoprimed with and without Mel than from NT ones with the lowest values obtained in OMel roots. Similar profile could be also observed in shoots and Kn-0 OMel shoots had lower SOD activity under stress conditions than Can-0 (Fig. 5G). After 7 d of NaCl treatment the activities of SOD were generally higher than at 24 h. Kn-0 roots grown from O and OMel seeds had significantly lower SOD activities under salt stress conditions than the corresponding Can-0 roots while this was not the case in NT variants (Fig. 5F). In leaves, similar observations were done (Fig. 5H).

#### Catalase

The activities of this enzyme in roots were lower than in leaves, they were generally higher under salt stress than control conditions and, except OMel variant in Can-0 roots grown under long-term stress conditions, not induced by seed treatment (Fig. 5I-L). In both ecotypes, the CAT activities in roots at 24 h were only slightly induced by salt stress and kept on the similar levels between variants with the exception of the OMel variant in Can-0 where the activities were comparable between conditions and the lowest (Fig. 5I). A very similar profile was also observed in leaves where the activities were approx. three times greater than in roots and were higher in all variants under stress conditions in both Can-0 and Kn-0 (Fig. 5K). After 7 d of stress the activities significantly increased in roots, especially under stress conditions, but in Kn-0 they were still comparable between plant variants while in Can-0 the effects of osmopriming were clearly visible (Fig. 5J). CAT activity was lower in the O variant in comparison with NT but much higher in OMel roots under stress conditions. In leaves, such induction in Can-0 was not observed in OMel variant upon one week of stress while in Kn-0 the activities were also induced by salt stress, were generally higher than in Can-0, with the highest value measured in the OMel variant (Fig. 5L).

*Guaiacol peroxidase*: Different POX activity patterns were observed in organs of both ecotypes after 24 h and 7 d of stress with much higher values measured in roots than in shoots and several-folds higher values in Kn-0 than in Can-0 roots (Fig. 5M-P). In O and OMel roots, POX activities in Kn-0 at 24 h were significantly higher under salt stress conditions in opposition to NT variant where its activity was lower than in unstressed plants (1.7 and 1.55 versus 0.95 µmol guaiacol/min/mg_PROT_, respectively) (Fig. 5M). POX activities were similar between variants in shoots upon 24 h of stress without any particular effect of seed treatment but the highest activities were present in Kn-0 variants grown under stress conditions (Fig. 5O). Upon one week of salt stress the POX activities raised very strongly in O and OMel Kn-0 roots which indicates the positive effect of seed treatment. Their activities were more than 5 times higher than in NT variant and reached the values of 3.31 and 3.74 versus 0.61 µmol guaiacol/min/mg_PROT_, respectively (Fig. 5N). Interestingly, the effect of osmopriming was manifested by a similar tendency as at 24 h: POX activities in O and OMel Kn-0 roots were lower than in the NT variant under control conditions (Fig. 5N). Although the activities in shoots were much lower than in roots, O and OMel Kn-0 variants exhibited the highest activities upon long-term salt stress treatment (Fig. 5P).

#### Ascorbate peroxidase

In opposition to POX, the activities of APX in roots were neither strongly induced by salt nor by seed osmopriming upon 24 h of 100 mM NaCl stress (Fig. 5Q). Moreover, APX activities slightly decreased in OMel variants of both ecotypes in both conditions in comparison with NT, probably due to the activity of Mel. In shoots, APX activity was around two times lower than in roots with no induction by salt and comparable values among variants of both ecotypes, except slight increase in the O variant of Kn-0 (Fig. 5S). After one week, the activities of APX in roots recovered in both ecotypes in the O and OMel variants but only in the OMel variants these activities were higher under stress than under control conditions (Fig. 5R). APX enzyme does not seem to be (naturally) implicated in NaCl stress response of these two ecotypes as the activities were lower under stress than under control conditions in NT and O variants. APX activities in shoots were much lower than in roots but still only in the OMel variants of both ecotypes the activities were higher under stress than under control conditions (Fig. 5T).

#### Glutathione peroxidase

After 24 h of 100 mM NaCl stress the activity of GSH-PX was stronger in roots than in shoots and, similarly to APX, GSH-PX in both organs was neither induced by salt stress nor by seed osmopriming with the exception of OMel variant of Can-0 roots where slight increase in GSH-PX activity under salt stress conditions was detected (Fig. S7A-D). GSH-PX activities in Kn-0 roots obtained from O and OMel seed variants were lower than in NT variants under both conditions. Activities in shoots at 24 h of stress were low and comparable between variants within each ecotype but the values measured under stress conditions were lower or equal to control conditions (Fig. S7C). After one more week of growth, the activities of GSH-PX in roots recovered in both ecotypes in all three variants but only in the OMel variants these activities were significantly higher under stress than under control conditions (Fig. S7B). In shoots, the highest GSH-PX activities were observed in O variants in both ecotypes and under both growth conditions and lower values were observed in all Kn-0 variants of NaCl-stressed plants than in unstressed ones (Fig. S7D).

#### Glutathione reductase

Different GSSG-R activity patterns were observed in both ecotypes (Fig. S7E-H). At 24 h the GSSG-R activities in Can-0 were significantly induced by stress in NT and O roots while in OMel variant they were similar between control and stressed plants, which indicates that these plants did not restore the pool of GSH upon short-term salt stress treatment (Fig. S7E). On the contrary, Kn-0 roots grown from seeds osmoprimed with and without Mel activated GSSG-R after 24 h of salt stress. In shoots, the GSSG-R activities in Can-0 had the same profile as in roots while in Kn-0 the activities in O and OMel variants were lower than in NT with slightly higher values in stressed plants (Fig. S7G). After 7 d of stress, the GSSG-R activities in Can-0 roots slightly recovered in NT while in OMel variant they rose around two times but the activity was only slightly higher in stressed than unstressed plants (Fig. S7F). The activities greatly recovered in Kn-0 after 7 d of stress treatment however, they were higher under stress conditions in NT and O variants but lower in OMel variant. Interestingly, in leaves, GSSG-R activities were lower in Can-0 O and OMel variants than in NT in both conditions with the lowest value in OMel grown under NaCl stress while the opposite situation was measured in Kn-0 OMel variant grown under the same condition where GSSG-R activity was the highest (Fig. S7H).

### Ethylene production

**Fig. 6:**
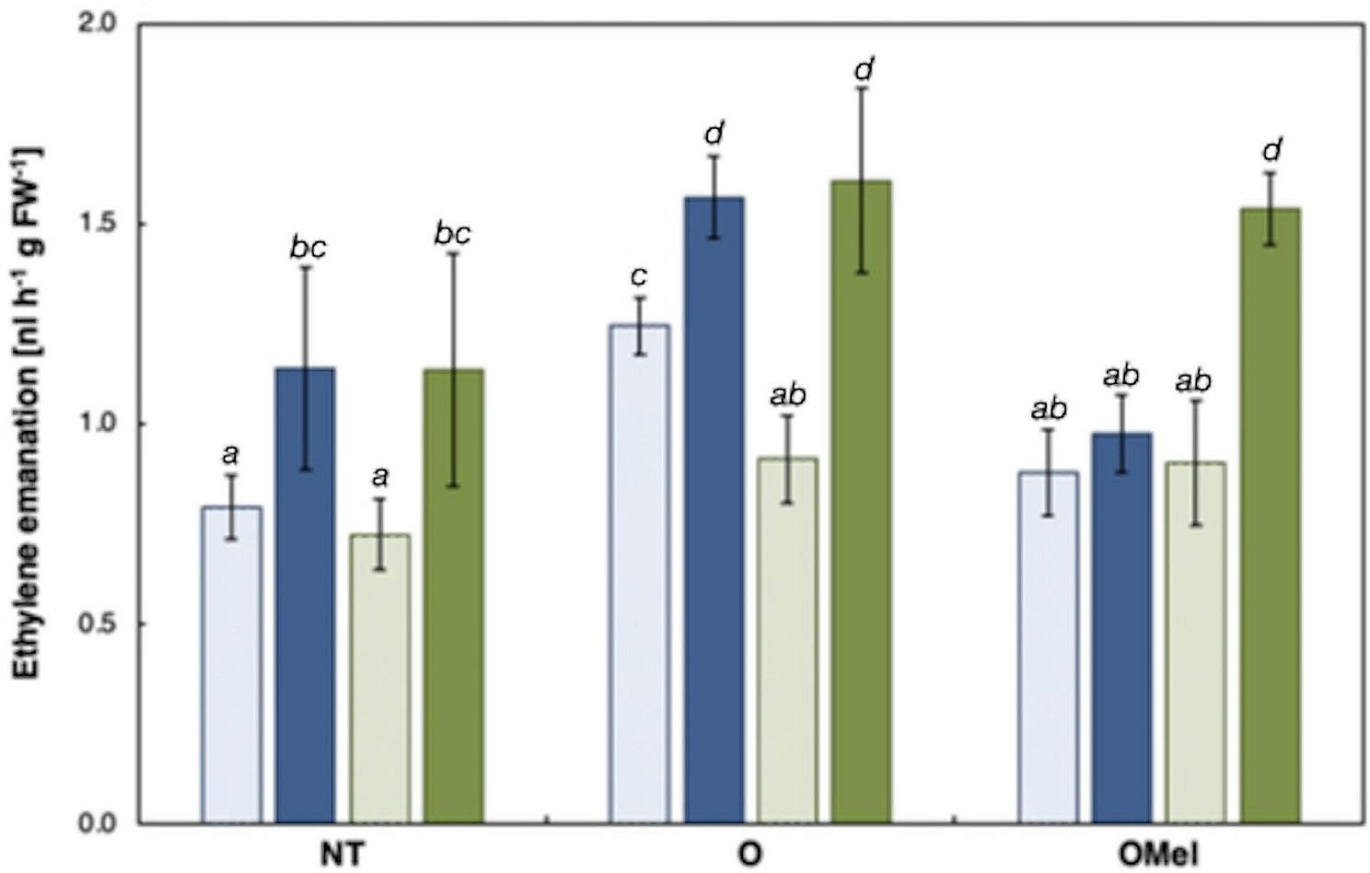
Ethylene emanation by in vitro-grown *Arabidopsis thaliana* Can-0 (blue) and Kn-0 (green) ecotypes. Two-week-old plants, grown from non-treated (NT), osmoprimed (O) and osmoprimed with melatonin (OMel) seeds, were transferred onto MS/2 medium (control, light bars) or the same medium supplemented with 100 mM NaCl (stress, coloured bars). Measurements were performed 24 h after transfer. Data represent mean values of three biological repeats (*n* = 3–4) ± SD. Values representing statistically significant differences at the 5% level (Duncan’s *post-hoc* test) are marked with lowercase letters

The ethylene production was measured during the day in plants that were previously transferred onto the control or 100 mM NaCl-containing MS/2 medium for 24 h, and normalized by the total fresh biomass. The emanation of ethylene was increased by salt stress in all plants measured, except in Can-0 plants grown from OMel seeds (Fig. 6). No difference in concentrations between the two ecotypes was observed, except in two cases: (i) Kn-0 plants grown from osmoprimed seeds produced less ethylene under control conditions than corresponding Can-0 plants, and (ii) Can-0 plants grown from OMel seeds did not show any ethylene synthesis induction under salinity stress. With this one exception, it is interesting to note that seed osmopriming increased to the similar extent ethylene production in salt stressed plants (Fig. 6).

## Discussion

### Seed osmopriming with the addition of Mel confers a higher stress resistance to salt stress

The phenotypic variation observed among natural *Arabidopsis thaliana* populations reflects the genetic variation that is necessary for their adaptation to specific growth habitats. A significant variation was already described for numerous developmental and adaptive traits including tolerance of abiotic stresses, such as drought, salinity, freezing or supply of mineral nutrients [85–89].

Salt stress tolerance and variability of RSA in response to salinity stress across *Arabidopsis thaliana* natural populations was previously studied without the influence of seed priming treatment [13, 14, 88, 90]. The aim of this work was to study the natural variation of biomass production and root architecture across an *Arabidopsis thaliana* core collection, which maximizes the genetic diversity of the species [91], in response to the pre-sawing seed treatment by osmopriming with and without Mel. Since the beneficial effects of seed priming combined with the use of biostimulators are visible especially under adverse environmental conditions, we analyzed the variability among plants grown under salt stress conditions. To defeat the limitations caused by salinity it is important to enhance plant stress resilience. This can be achieved by breeding programmes, genetic engineering technologies or genome modification approaches but also by generating plant stress memory by pre-sawing seed treatment [24].

There are many ways in which Mel can be applied to plants, as irrigation supplement, as a foliage spraying component, as a seed-coating-reagent, or it can be also added into the culture medium. Additionally, it can be included in the seed imbibition solution just before sawing. In our laboratory, we have developed a method for applying Mel during pre-sowing seed treatment through osmo- or hydropriming [49, 55, 74] that was used in several species and was also applied in the present study. Mel was shown to be an excellent biostimulator candidate [92] and Mel supplementation via seed priming is a very promising method not only to improve germination but also to further protect the seedlings growing under adverse environmental conditions [47, 62, 64, 65, 93]. Why it is beneficial to add Mel during seed priming? Firstly, the timing of biostimulator application is crucial, and it should align with pivotal stages of plant development like for instance germination and seedling establishment. Secondly, seed priming imparts greater stress tolerance, as seeds subjected to the pre-sawing treatment, in addition to the initial stress exposure, are also developmentally more advanced toward complete germination than non-treated seeds. In this case, priming memory is induced by two events and the presence of a biostimulator added during seed treatment further enhances seedlings’ ability to cope with adverse environmental conditions. This ensures better protection for growing seedlings from the moment of germination. Thirdly, Mel activates the ROS scavenging system in germinating seeds. Finally, as demonstrated by Kołodziejczyk et al. [94] in maize, seed priming with Mel induced synthesis of a new set of proteins in embryonic axes. The majority of these additional proteins are related to defense, anti-stresses mechanisms and detoxification but they also belong to the energy metabolism as well as proteins involved in proteome plasticity, improving protein synthesis, folding and storage. In comparison with the unprimed seeds, plants grown from the pre-treated seeds quickly and efficiently adapt to the changing and stressful environmental conditions.

### Seed osmopriming modifies root architecture

The comparison of RSA parameters between NT and OMel plant variants showed that Mel can improve the measured traits in plants cultivated under stress conditions. In their studies, Guo et al. [62] and Khan et al. [64] demonstrated in wheat and rapeseed plants, respectively, that seed priming with Mel led to a greater enhancement of root growth under drought stress in comparison with non-primed plants, as opposed to control conditions. In our research, we observed a similar trend in certain *Arabidopsis thaliana* ecotypes regarding biomass production, PR length or N_LR_. The overall seed osmopriming with and without Mel significantly improved measured traits in most of analyzed ecotypes under both, control and salt stress conditions, with the greatest performance observed predominantly in plants grown from seeds osmoprimed with the addition of Mel. However, some ecotypes exhibited different behaviors. In contrast to the positive effects on growth observed with exogenously applied Mel, as described in numerous communications, we also found some ecotypes, including Can-0 and Sakata, showing negative effects of pre-sawing seed treatment with and without Mel, as manifested by reduced biomass, PR length and N_LR_. The habitats from which these ecotypes originate and specific adaptation traits might be responsible for such phenotype.

RSA is highly sensitive to changes in its environment including the availability of mineral resources and water or intensities of abiotic stresses such as drought and salinity. Therefore, the plasticity of root traits is, among others, responsible for salt stress tolerance of a plant. Indeed, Julkowska et al. [13] identified in their studies across *Arabidopsis thaliana* accessions that PR length as well as the number and length of LRs are three RSA parameters that are most responsive to salt stress. Our analysis of those parameters showed high variability in responsiveness of ecotypes to salinity stress. Julkowska et al. [13] calculated the relative effect of salt on growth rates of RSA parameters to subsequently categorize the *Arabidopsis thaliana* accessions by using Ward’s hierarchical cluster method. Accordingly, they revealed four different root morphological strategies (I, II, III and IV) where accessions belonging to groups I – III were less affected by salt stress than those from group IV that were characterized by the most affected phenotypes (shorter PRs and LRs but also reduced N_LR_). In their analysis Can-0 ecotype, which we identified as one of the least responsive to seed osmopriming treatments, clustered within group IV [13].

### Seed osmopriming supplemented with Mel modulates H_2_O_2_ accumulation

Stress memory, established during seed priming, leads to the expression of favorable traits that improve stress tolerance and alleviate the detrimental effects of drought and salinity, among others, reducing the level of oxidative stress in germinating seeds and growing seedlings. In both analyzed ecotypes, upon short-term (24 h) salt stress, the amounts of H_2_O_2_ produced by roots grown from osmoprimed seeds were lower than those produced by roots obtained from the NT seeds, indicating the positive effect of osmopriming on redox status. This effect was particularly notable in plants grown from seeds osmoprimed with the addition of Mel, which itself acts directly as a ROS scavenger. Indeed, Mel has been shown to protect plants from oxidative stress and the positive effects of seed priming, and seed priming with Mel, on H_2_O_2_ content in plants grown under drought or salt stress conditions have been reported by several authors [29, 62, 64–66, 95, 96].

ROS on one hand can be harmful to cellular components and metabolic pathways, and on the other hand, act as signaling molecules to trigger stress responses [97]. It is possible that the accumulation of H_2_O_2_ at 24 h, which was higher than after long-term (7 d) salt treatment, acts as signal to activate the defense systems in roots and shoots. In fact, upon 24 h of salt treatment more H_2_O_2_ was accumulated under stress conditions than under control conditions in roots of NT, O and OMel plants whereas SOD activities that generate H_2_O_2_ were lower under stress conditions in all variants of both ecotypes. Additionally, in shoots, the amount of H_2_O_2_ accumulated by Kn-0 upon short-time stress treatment was more than two times higher than that in Can-0 in all plant variants while the activities of SOD were rather comparable between the accessions among all three variants as well as the activities of other H_2_O_2_-scavenging antioxidant enzymes were lower at this time point. Interestingly, after two weeks of culture the amount of H_2_O_2_ was higher in OMel Kn-0 roots than in the NT ones grown under both control and stress conditions which pinpoints the beneficial role of osmopriming with Mel on H_2_O_2_ signaling. A similar profile was also observed in Kn-0 shoots which suggests that, in opposition to Can-0, a higher level of H_2_O_2_ may act as a signal for an acclimatory response to stress in this ecotype, to induce antioxidant response for instance, which in turn might account for a better growth of Kn-0 plants under stress conditions [98, 99]. Indeed, the comparable activities of SOD in Kn-0 roots between variants, and even lower SOD activities in OMel shoots than in O variant, grown for one week under salt stress conditions might support such possibility.

### Osmopriming with Mel changes the activity of the enzymatic antioxidative system

The cellular levels of ROS are regulated by an array of antioxidant enzymes, the activities of which are enhanced by oxidative stress. Proteomic analysis of salt-stressed alfalfa and *Arabidopsis* roots demonstrated the upregulation of antioxidant enzymes [100, 101]. In our work, enhanced activities were also observed in Can-0 and Kn-0 ecotypes. However, not all enzymes showed enhanced activities, and the extent of enhancement varied depending on the duration of the salt stress treatment or the seed priming variant. This observation confirms the involvement of distinct enzymes against salinity-induced oxidative stress. With the exception of the robustly upregulated POX in Kn-0, the activities of the measured enzymes were mildly induced by seed osmopriming upon 24 h of salt stress. Subsequently, the activities increased significantly in both organs over the next few days. According to microarray data [102], the transcripts for SOD genes were downregulated in *Arabidopsis* roots in response to short-term salt treatment. Interestingly, in both ecotypes, the activities of SOD at 24 h of salt stress were the highest in roots of the NT variant and the lowest in roots of the OMel variant, which correlated well with low H_2_O_2_ contents in roots from osmopriming treatments with Mel. These data provide insights into the impact of seed osmopriming on SOD activity and the role of exogenous Mel as radical scavenger in young plants exposed to stress. In both ecotypes, SOD activities increased after one week of growth under stress conditions but this increase was most pronounced in Can-0, especially in plants from osmoprimed seeds, suggesting higher responsiveness and/or more elevated oxidative stress in this ecotype. These activities in Can-0 corresponded with TBARS-evaluated lipid peroxidation. In contrast, although in Kn-0 SOD activities were slightly higher in both organs from O and OMel seeds than in the NT ones, they were rather comparable between variants while lipid peroxidation levels were much more lower in O and OMel Kn-0 organs suggesting lower oxidative stress in this ecotype, even under salinity conditions. This is most probably due to the protective role of osmopriming, exogenous Mel and activity of other antioxidant enzymes. In fact, Can-0 and Kn-0 ecotypes greatly differed in term of POX involvement in their acclimatory responses. In opposition to Can-0, an important induction of POX was measured in both organs in Kn-0 O and OMel variants at 24 h of stress and very strong induction after one week of stress in these variants, with the highest value recorded in roots obtained from OMel seeds. POX in Kn-0 roots were found to be the most responsive to stress among all analyzed enzymes and activated in plants grown from osmoprimed seeds which may explain lower lipid peroxidation, lower H_2_O_2_ content and a better root growth of the OMel variant of this ecotype than that of the NT one under stress conditions. Osmopriming- and Mel-induced enhanced activities of POX in roots might confer higher stress resistance to Kn-0 seedlings. Lower H_2_O_2_ contents and lipid peroxidation and enhanced activities of principal antioxidant enzymes, including SOD, POX, APX and CAT are, among others, common features of Mel effects, previously reported in germinating seeds [40, 66], plants grown from seeds primed with the addition of Mel [47, 62, 64, 65, 103] and in plants treated with Mel during their development [39, 41–44, 63, 70] grown under drought or salt stress conditions. It is well documented that priming with Mel alleviated adverse effects of drought and salinity by intensifying ROS scavenging. However, our work clearly showed that not all antioxidant enzymes were activated by seed osmopriming or by salt treatment. For instance, CAT were much less responsive than POX and the impact of osmopriming with the addition of Mel was almost not observed with the exception of roots of Can-0 OMel variant after one week of stress. The APX activity was not induced in roots and shoots of NT and O variants of both ecotypes, either upon short-term or long-term stress treatment, indicating that APX was not implicated in salt stress response in these two ecotypes. Similar results were reported by Hernandez et al. [104] in *Brassica oleracea* roots and by Yu et al. [105] in *Brassica napus* leaves where the APX activities were decreased or comparable to the control ones under long-term salt stress treatment, respectively. Interestingly, APX activities in roots and shoots of OMel variants upon long-term stress treatment were higher in both ecotypes under salinity stress than under control conditions, which once again pinpoints the positive stimulation of the antioxidant enzymes by Mel added to the osmoprimer. Finally, GSH-PX were induced by osmopriming with Mel in Can-0 roots upon short-time and long-time NaCl treatments while in Kn-0 roots only at long-time NaCl treatment.

In view of the observed results, seed osmopriming with and without Mel reinforced the activities of some of the assayed enzymes but an ecotype-specific responsiveness to salt stress and osmopriming treatment was clearly observable. Enhanced by osmopriming with Mel, the activities of CAT, POX, APX and GSH-PX protected better Kn-0 organs against oxidative stress than the enzymes active in the Can-0 organs.

### Osmopriming with Mel modifies Na/K ratio, proline accumulation and ethylene production

As observed in most species exposed to salinity stress, there is an increase in sodium content with a concomitant reduction of potassium uptake, leading to impaired ionic balance [1]. Accordingly, sensitivity to salt stress was associated with the inability to maintain high K^+^ concentrations and low Na^+^/K^+^ ratio in the cytoplasm [106]. While our mineral analysis confirmed these tendencies in both ecotypes, some ecotype-specific differences could be observed, particularly in relation to seed osmopriming with Mel. Roots and shoots of the NT plants of the salt sensitive ecotype (Can-0) accumulated significantly more sodium than those of the Kn-0. In contrast to Kn-0, potassium concentrations significantly decreased in NT Can-0 salt-stressed roots in comparison with the unstressed ones, and were also lower in salt-stressed Can-0 OMel roots and shoots than in Kn-0 plants. These differences translate into higher Na/K ratios in salt-stressed Can-0 roots and shoots of nearly all variants, suggesting better adaptation of the Kn-0 ecotype and the protective effect of osmopriming with Mel.

The synthesis of ethylene is activated by numerous abiotic stresses and ethylene-mediated responses are part of the adaptive strategies that enable plants to survive under adverse environmental conditions [107]. Ethylene has been shown to enhance salt stress tolerance by regulating the Na^+^/K^+^ ratio, maintaining nutrient homeostasis and activating antioxidant defense mechanisms [108, 109]. Our measurements showed that ethylene production was induced by salt stress in both analyzed ecotypes but was stronger in plants derived from osmoprimed seeds, except in OMel Can-0 plants. Similarly, Lechowska et al. [110] reported salt-induced ethylene synthesis in *Brassica napus* seedlings grown from osmoprimed seeds. Seed treatment with the addition of Mel enhanced salt stress tolerance by promoting ethylene synthesis in grapevine and wheat [111, 112]. Accordingly, the higher salt sensitivity of Can-0, exhibiting reduced root growth and oxidative stress response, as well as strong proline accumulation in both organs, could also be related to lower ethylene synthesis. In support, Lv et al. [113] observed that transgenic Arabidopsis plants overproducing proline under heat stress conditions were characterized by lower survival rate and by impaired ethylene biosynthesis than plants that did not accumulate proline.

Proline has been proposed to play a role in osmotic adjustment, protection of membrane integrity and protein stabilization but also in ROS scavenging and maintenance of redox homeostasis [83]. Although proline was intensively accumulated after one week of growth under 100 mM NaCl by both ecotypes, the pre-sawing seed treatment further enhanced the synthesis of this osmolyte in both roots and shoots, but to different extents in Can-0 and Kn-0, suggesting an ecotype-specific responsiveness. The synthesis of proline in Can-0 ecotype, especially in leaves, was even stronger when Mel was added during seed osmopriming, which was not the case in Kn-0. The studies in different species underline the positive impact of Mel treatment, either on seeds or plants, on the accumulation of proline under drought stress [40, 42, 64, 65, 114]. It is possible that in Can-0 the proline accumulation induced by osmopriming with Mel may be involved in ROS scavenging, since the activities of antioxidant enzymes, were lower in comparison with Kn-0. Indeed, the lack of enhanced CAT and POX activities under long-term stress treatment, together with the TBARS levels, indicating a higher level of oxidative stress in this ecotype than in Kn-0, especially in leaves, suggest that proline may play a role of ROS scavenger in Can-0 organs exposed to long-term stress. It is also possible that proline protected photosynthetic and antioxidant enzymes activities under salt stress conditions [115]. The strong accumulation of proline in Can-0 plants obtained from osmoprimed seeds may in turn result in reduced growth of organs under both control (priming is perceived as a stress factor) and salt stress conditions. In fact, although plants can differ in the amount of proline accumulated under stress conditions and the link between proline accumulation and stress tolerance is not clear, several studies underline a relationship between growth and overproduction of osmolytes as an adaptive response to osmotic stress. Karakas et al. [116] found that transgenic tobacco overproducing mannitol showed greater salt tolerance but was characterized by slower growth. Similar observation was made in transgenic tobacco overexpressing trehalose-6-phosphate synthase [117]. Maggio et al. [118] observed that in proline-overproducing transgenic yeast, the higher the intracellular level of proline, the more their growth was reduced. Such strategy of adaptation may be employed by Can-0 plants grown from seeds osmoprimed with Mel to withstand the imposed salt stress. Thus, osmopriming-induced proline synthesis under salt stress conditions might result in growth and biomass reduction. In contrast, in Kn-0 OMel variant, the level of oxidative stress seemed lower, and the growth of organs could be maintained.

### Osmopriming enriched with Mel differentially enhances salt stress response in Can-0 and Kn-0 ecotypes

The levels of endogenous Mel in species and tissues are variable under different environmental conditions. Plants grown under moderate conditions have lower Mel content than those exposed to changing or harmful conditions. Stress tolerant genotypes have generally higher Mel levels than sensitive ones [119, 120] and exposure to stresses like irradiation or salinity is known to increase endogenous Mel contents [121–124]. The authors conclude that Mel-rich species can better cope with stress since Mel and its metabolites protect tissues from oxidative damages. The variations in Mel contents are particularly related to specific habitats and altitude and reflect ecological adaptations. Indeed, alpine and Mediterranean species or varieties, which are growing on higher altitudes and are exposed to high natural light and UV radiation as well as to temperature fluctuations are characterized by higher contents of endogenous Mel than the same or related species from other habitats [125–127]. We therefore quantified the endogenous Mel levels in three variants of seeds of all the accessions used in this experiment. We found that NT seeds of Can-0 contained more endogenous Mel (2.4 ng g^− 1^ FW) than the NT seeds of Kn-0 (0 ng g^− 1^ FW) which is certainly related to altitude and arid environment – the volcanic islands, form which Can-0 accession originates (Fig. S8). According to The Arabidopsis Information Resource, Can-0 ecotype was collected on high altitude habitat (1260 m) whereas Kn-0 ecotype comes from Lithuanian lowland habitat (1–100 m) (http://www.arabidopsis.org). The levels of endogenous Mel after osmopriming treatment in Can-0 and Kn-0 seeds reached 1.4 and 0.6 ng g^− 1^ FW, respectively, confirming that osmopriming, as a stress factor, raises Mel levels, especially in Can-0. In contrast, concentrations in OMel seeds were not significantly different, 434 ng g^− 1^ FW in Can-0 and 469 ng g^− 1^ FW in Kn-0, which indicates that the effect of exogenous Mel on the growth phenotype and differential induction of antioxidant enzymes and proline synthesis was not correlated with its initial level in seeds.

The pre-treatment with Mel was previously shown to increase the tolerance to drought stress in both, tolerant and sensitive species (or varieties), but more profound effects were observed in sensitive species [41, 103]. It was also demonstrated that plant species (or varieties) with higher salt stress tolerance are characterized by higher activities of antioxidant enzymes, and thus lower H_2_O_2_ contents and lipid peroxidation, than the sensitive species (or varieties) [128–130]. On the other hand, salt-sensitive varieties frequently accumulated more proline in their leaves than the tolerant ones [131–133]. Seed osmopriming with and without Mel resulted in differential responsiveness and induction of different protective mechanisms in Can-0 and Kn-0. The treatments had opposite effects on the level of oxidative stress in the two ecotypes, adapted to different habitats, and conferred higher salt stress tolerance to Kn-0 which appeared to be more responsive to osmopriming and osmopriming with Mel. Although, a direct correlation cannot always be traced between salt stress tolerance and the activation of antioxidant enzymes by osmopriming in both analyzed ecotypes, our results suggest that Kn-0 could be protected by POX. Thus, the improved growth in Kn-0 under salinity stress seems to be associated with a better protection against oxidative stress. On the contrary, although seed osmopriming with Mel induced the activities of some antioxidant enzymes in Can-0 grown under long-term stress conditions, their activities do not seem sufficient to efficiently detoxify ROS and protect plants against oxidative damage as evidenced by higher lipid peroxidation, proline accumulation and increased salt sensitivity.

Understanding environment-induced modifications of growth parameters for plant survival, development and productivity is one of the most important challenges of modern agriculture. Thus, the use of model species, offering invaluable genetic resources, can be beneficial in term of identification of mechanisms that confer higher tolerance to different types of stresses. Indeed, the two isolated contrasted *Arabidopsis thaliana* ecotypes constitute a starting point to identify the genetic factors controlling efficiency of seed osmopriming treatment. Additionally, studying the protective role of biostimulators like Mel and identifying the physiological processes they improve may be valuable for obtaining plants that are better adapted to changing environment conditions.

## Conclusion

In both ecotypes exposed to salinity stress, the effects of osmopriming and osmopriming with Mel were clearly observed. However, in Can-0 and Kn-0 growth and antioxidant responses differed. They adopted different growth strategies; osmopriming- and Mel-induced reduction of RSA parameters was observed in Can-0 while the opposite tendency was observed in Kn-0. In Kn-0, both seed osmopriming and Mel induced a lower root sensitivity to oxidative stress whereas Can-0 roots grown from seeds subjected to osmopriming treatments experienced higher oxidative stress.

Plant tolerance to higher concentrations of NaCl is controlled by many different mechanisms [1]. Among them, the effective control of the oxidative damage induced by osmotic stress and ion toxicity is critical for salt tolerance. In view of the results obtained in our analyzes, the antioxidative system was significantly affected by salt and induced by osmopriming treatments but in a different manner and to a different extent in the two studied ecotypes. This study is the first step to the identification of molecular mechanisms underlying the contrasting response to osmopriming and exogenous Mel.

### Electronic supplementary material

Below is the link to the electronic supplementary material.

Supplementary Material 1

Supplementary Material 2

Supplementary Material 3

Supplementary Material 4

Supplementary Material 5

Supplementary Material 6

Supplementary Material 7

Supplementary Material 8

Supplementary Material 9

Supplementary Material 10

## Data Availability

No datasets were generated or analysed during the current study.
