# Supplementary material for: An ecotype-specific effect of osmopriming and melatonin during salt stress in Arabidopsis thaliana
Source: BMC Plant Biol. 2024 Jul 25;24:707. doi: 10.1186/s12870-024-05434-5 (PMC11270801; doi:10.1186/s12870-024-05434-5)
Supplement: Supplementary file 1 — Supplementary Material 1 [file 12870_2024_5434_MOESM1_ESM.docx]

**Legends to supplementary figures**

**Fig. S1** Delimitation and terminology of the distinct zones of the primary root for morphological trait calculations. The root zones were determined by the position of the first and the last lateral root including the possibilities with none, one and several lateral roots. The following zones (Z) were defined: Z1, primary root (PR); Z2, branched zone; Z3, basal zone; Z4, apical zone. Root architecture traits were calculated by RootNav software.

**Fig. S2.** Total biomass of 30 *Arabidopsis thaliana* ecotypes used in the screening. Seedlings were grown from non-treated (NT), osmoprimed (O) and osmoprimed with melatonin (OMel) seeds on vertical plates on control MS/2 medium and transferred 5 days after germination onto the same medium supplemented with or without 100 mM NaCl. 7 days after transfer and following analysis of root system architecture, shoots and roots were pooled and weighed. Data represent mean values of 3 biological repeats (*n*= 15) ± SD. Statistical significance at the 5% level (Duncan's *post-hoc* test) was determined individually within each ecotype grown under control and salt stress conditions.

**Fig. S3.** Primary root length (L_PR_) of 30 *Arabidopsis thaliana* ecotypes used in the screening. Seedlings were grown from non-treated (NT), osmoprimed (O) and osmoprimed with melatonin (OMel) seeds on vertical plates on control MS/2 medium and transferred 5 days after germination onto the same medium supplemented with or without 100 mM NaCl. Length of primary root measurements were taken 7 days after transfer. Data represent mean values of 3 biological repeats (*n*= 10-15) ± SD. Statistical significance at the 5% level (Duncan's *post-hoc* test) was determined individually within each ecotype grown under control and salt stress conditions.

**Fig. S4.** Number of lateral roots (N_LR_) of 30 *Arabidopsis thaliana* ecotypes used in the screening. Seedlings were grown from non-treated (NT), osmoprimed (O) and osmoprimed with melatonin (OMel) seeds on vertical plates on control MS/2 medium and transferred 5 days after germination onto the same medium supplemented with or without 100 mM NaCl. Number of lateral roots counting were taken 7 days after transfer. Data represent mean values of 3 biological repeats (*n*= 10-15) ± SD. Statistical significance at the 5% level (Duncan's *post-hoc* test) was determined individually within each ecotype grown under control and salt stress conditions.

**Fig. S5.** Sum of lateral root lengths (ΣL_LR_) of 30 *Arabidopsis thaliana* ecotypes used in the screening. Seedlings were grown from non-treated (NT), osmoprimed (O) and osmoprimed with melatonin (OMel) seeds on vertical plates on control MS/2 medium and transferred 5 days after germination onto the same medium supplemented with or without 100 mM NaCl. Length of lateral roots measurements were taken 7 days after transfer. Data represent mean values of 3 biological repeats (*n*= 10-15) ± SD. Statistical significance at the 5% level (Duncan's *post-hoc* test) was determined individually within each ecotype grown under control and salt stress conditions.

**Fig. S6** Root architecture comparison of two contrasted Can-0 and Kn-0 ecotypes in response to salt stress. Seedlings were grown from non-treated (NT), osmoprimed (O) and osmoprimed with melatonin (OMel) seeds on vertical plates on control MS/2 medium and transferred 5 days after germination onto the same medium supplemented with or without 100 mM NaCl for one more week. Pictures were exported from the RootNav software. Bar, 1 cm.

**Fig. S7** Glutathione peroxidase (GSH-PX) and reductase (GSSG-R) activities in *Arabidopsis* Can-0 (blue) and Kn-0 (green) ecotypes. Roots (A, B, E, F) and shoots (C, D, G, H) of one-week-old plants, grown from non-treated (NT), osmoprimed (O) and osmoprimed with melatonin (OMel) seeds, were harvested separately 24 h (A, C, E, G) or 7 days (B, D, F, H) after transfer onto MS/2 medium (control, light bars) or the same medium supplemented with 100 mM NaCl (stress, coloured bars). Data represent mean values of three biological repeats (*n*= 6-9) ± SD. Values representing statistically significant differences at the 5% level (Duncan's *post-hoc* test) are marked with lowercase letters.

**Fig. S8.** Melatonin quantification in three variants of seeds of 30 *Arabidopsis thaliana* ecotypes. The following variants of seeds were used: non-treated (A), osmoprimed (B) and osmoprimed with melatonin (C). Data represent mean values of 3 biological repeats (*n*= 9) ± SD. Values representing statistically significant differences at the 5% level (Duncan's *post-hoc* test) are marked with lowercase letters.
