# Supplementary material for: An ecotype-specific effect of osmopriming and melatonin during salt stress in Arabidopsis thaliana
Source: BMC Plant Biol. 2024 Jul 25;24:707. doi: 10.1186/s12870-024-05434-5 (PMC11270801; doi:10.1186/s12870-024-05434-5)
Supplement: Supplementary file 2 — Supplementary Material 2 [file 12870_2024_5434_MOESM2_ESM.docx]

**Statistics - ANOVA results**

**Table 1: Roots – ANOVA results:**

***Sodium* –** Ecotypes _(Can. Kn)_ F_(1; 26)_=3.59, *p*=0.07; Variants _(NT. O. OMel)_ F_(2; 26)_=19.52, *p*<0.00001; Conditions _(C. NaCl)_ F_(1; 26)_=671.90, *p*<0.000001; interaction Ecotypes x Variants F_(2; 26)_=1.18, *p*=0.32; interaction Ecotypes x Conditions F_(1; 26)_=3.11, *p*=0.09; interaction Variants x Conditions F_(2; 26)_=15.14, *p*<0.00005; interaction Ecotypes x Variants x Conditions F_(2; 36)_=1.12, *p*=0.34.

***Potassium* –** Ecotypes _(Can. Kn)_ F_(1; 26)_=2.30, *p*=0.14; Variants _(NT. O. OMel)_ F_(2; 26)_=31.13, *p*<0.000001; Conditions _(C. NaCl)_ F_(1; 26)_=0.94, *p*=0.34; interaction Ecotypes x Variants F_(2; 26)_=10.16, *p*<0.001; interaction Ecotypes x Conditions F_(1; 26)_=1.22, *p*=0.28; interaction Variants x Conditions F_(2; 26)_=3.04, *p*=0.065; interaction Ecotypes x Variants x Conditions F_(2; 36)_=12.90. *p*<0.0005.

***Ratio Na/K* –** Ecotypes _(Can. Kn)_ F_(1; 26)_=7.46, *p*<0.05; Variants _(NT. O. OMel)_ F_(2; 26)_=32.89, *p*<0.000001; Conditions _(C. NaCl)_ F_(1; 26)_=594.09, *p*<0.000001; interaction Ecotypes x Variants F_(2; 26)_=0.376, *p*=0.069; interaction Ecotypes x Conditions F_(1; 26)_=6.04, *p*<0.05; interaction Variants x Conditions F_(2; 26)_=25.61, *p*<0.000001; interaction Ecotypes x Variants x Conditions F_(2; 36)_=0.324. *p*=0.73.

**Shoots – ANOVA results:**

***Sodium* –** Ecotypes _(Can. Kn)_ F_(1; 24)_=8.44. *p*<0.01; Variants _(NT. O. OMel)_ F_(2; 24)_=1.03. *p*=0.37; Conditions _(C. NaCl)_ F_(1; 24)_=2358.67. *p*<0.000001; interaction Ecotypes x Variants F_(2; 24)_=4.51. *p*<0.05; interaction Ecotypes x Conditions F_(1; 24)_=8.01. *p*<0.01; interaction Variants x Conditions F_(2; 24)_=1.52. *p*=0.24; interaction Ecotypes x Variants x Conditions F_(2; 24)_=4.11. *p*<0.05.

***Potassium* –** Ecotypes _(Can. Kn)_ F_(1; 28)_=18.43. *p*<0.0005; Variants _(NT. O. OMel)_ F_(2; 28)_=0.58. *p*=0.57; Conditions _(C. NaCl)_ F_(1; 28)_=793.2. *p*<0.000001; interaction Ecotypes x Variants F_(2; 28)_=0.43. *p*=0.65; interaction Ecotypes x Conditions F_(1; 28)_=4.03. *p*=0.05; interaction Variants x Conditions F_(2; 28)_=2.07. *p*=0.14; interaction Ecotypes x Variants x Conditions F_(2; 28)_=2.50. *p*=0.10.

***Ratio Na/K* –** Ecotypes _(Can. Kn)_ F_(1; 24)_=21.99, *p*<0.0001; Variants _(NT. O. OMel)_ F_(2; 24)_=4.11, *p*<0.05; Conditions _(C. NaCl)_ F_(1; 24)_=1392.83, *p*<0.000001; interaction Ecotypes x Variants F_(2; 24)_=4.85, *p*<0.05; interaction Ecotypes x Conditions F_(1; 24)_=21.48, *p*<0.0001; interaction Variants x Conditions F_(2; 24)_=4.00, *p*<0.05; interaction Ecotypes x Variants x Conditions F_(2; 24)_=4.78. *p*<0.05.

**Figures**

**Fig. 3: Can-0, Biomass & RSA – ANOVA results: (A)** Variants _(NT, O, OMel)_ F_(2; 12)_=5.9, *p*<0.05; Conditions _(C, NaCl)_ F_(1; 12)_=1.42, *p*=0.26; interaction Variants x Conditions F_(2; 12)_=0.138, *p*=0.87. **(B)** Variants _(NT, O, OMel)_ F_(2; 63)_=15.93, *p*<0.000005; Conditions _(C, NaCl)_ F_(1; 63)_=254.63, *p*<0.0000001; interaction Variants x Conditions F_(2; 63)_=4.46, *p*<0.05. **(C)** Variants _(NT, O, OMel)_ F_(2; 63)_=11.17, *p*<0.0001; Conditions _(C, NaCl)_ F_(1; 63)_=47.72, *p*<0.0000001; interaction Variants x Conditions F_(2; 63)_=11.44, *p*<0.0001. **(D)** Variants _(NT, O, OMel)_ F_(2; 63)_=3.86, *p*<0.05; Conditions _(C, NaCl)_ F_(1; 63)_=106.22, *p*<0.0000001; interaction Variants x Conditions F_(2; 63)_=9.44, *p*<0.0005.

**Kn-0, Biomass & RSA – ANOVA results: (E)** Variants _(NT, O, OMel)_ F_(2; 12)_=68.52, *p*<0.0000005; Conditions _(C, NaCl)_ F_(1; 12)_=19.14, *p*<0.001; interaction Variants x Conditions F_(2; 12)_=3.37, *p*=0.07 **(F)** Variants _(NT, O, OMel)_ F_(2; 68)_=42.31, *p*<0.0000001; Conditions _(C, NaCl)_ F_(1; 68)_=175.08, *p*<0.000001; interaction Variants x Conditions F_(2; 68)_=7.39, *p*<0.05. **(G)** Variants _(NT, O, OMel)_ F_(2; 68)_=28.13, *p*<0.0000001; Conditions _(C, NaCl)_ F_(1; 68)_=185.43, *p*<0.0000001;; interaction Variants x Conditions F_(2; 68)_=9.66, *p*<0.0005. **(H)** Variants _(NT, O, OMel)_ F_(2; 68)_=116.05; *p*<0.0000001; Conditions _(C, NaCl)_ F_(1; 68)_=408.87; *p*<0.0000001; interaction Variants x Conditions F_(2; 68)_=42.34, *p*<0.0000001.

**Fig. 4: TBARS – ANOVA results:** (**A)** Ecotypes _(Can, Kn)_ F_(1; 108)_=34.36, *p*<0.000001; Variants _(NT, O, OMel)_ F_(2; 108)_=1.78, *p*=0.17; Conditions _(C, NaCl)_ F_(1; 108)_=106.18, *p*<0.000001; interaction Ecotypes x Variants F_(2; 108)_=22.74, *p*<0.000001; interaction Ecotypes x Conditions F_(1; 108)_=19.37, *p*<0.00005; interaction Variants x Conditions F_(2; 108)_=0.16, *p*=0.85; interaction Ecotypes x Variants x Conditions F_(2; 108)_=3.78, *p*<0.05. (**B**) Ecotypes _(Can, Kn)_ F_(1; 126)_=217.57, *p*<0.000001; Variants _(NT, O, OMel)_ F_(2; 126)_=14.60, *p*<0.000005; Conditions _(C, NaCl)_ F_(1; 126)_=51.58, *p*<0.000001; interaction Ecotypes x Variants F_(2; 126)_=67.12, *p*<0.000001; interaction Ecotypes x Conditions F_(1; 126)_=9.15, *p*<0.05; interaction Variants x Conditions F_(2; 126)_=4.39, *p*<0.05; interaction Ecotypes x Variants x Conditions F_(2; 126)_=12.23, *p*<0.00005.

**Proline – ANOVA results:** (**C)** Ecotypes _(Can, Kn)_ F_(1; 96)_=74.56, *p*<0.000001; Variants _(NT, O, OMel)_ F_(2; 96)_=118.93, *p*<0.000001; Conditions _(C, NaCl)_ F_(1; 96)_=2091.70, *p*<0.000001; interaction Ecotypes x Variants F_(2; 96)_=54.65, *p*<0.000001; interaction Ecotypes x Conditions F_(1; 96)_=12.13, *p*<0.000001; interaction Variants x Conditions F_(2; 96)_=80.06, *p*<0.000001; interaction Ecotypes x Variants x Conditions F_(2; 96)_=55.02, *p*<0.000001. (**D**) Ecotypes _(Can, Kn)_ F_(1; 96)_=147.09, *p*<0.000001; Variants _(NT, O, OMel)_ F_(2; 96)_=167.56, *p*<0.000001; Conditions _(C, NaCl)_ F_(1; 96)_=1322.42, *p*<0.000001; interaction Ecotypes x Variants F_(2; 96)_=101.74, *p*<0.000001; interaction Ecotypes x Conditions F_(1; 96)_=43.69, *p*<0.000001; interaction Variants x Conditions F_(2; 96)_=95.17, *p*<0.000001; interaction Ecotypes x Variants x Conditions F_(2; 96)_=127.63, *p*<0.000001.

**Fig. 5: H_2_O_2_ – ANOVA results:** (**A)** Ecotypes _(Can, Kn)_ F_(1; 36)_=110.31, *p*<0.000001; Variants _(NT, O, OMel)_ F_(2; 36)_=45.50, *p*<0.000001; Conditions _(C, NaCl)_ F_(1; 36)_=98.18, *p*<0.000001; interaction Ecotypes x Variants F_(2; 36)_=36.77, *p*<0.000001; interaction Ecotypes x Conditions F_(1; 36)_=11.99, *p*<0.005; interaction Variants x Conditions F_(2; 36)_=10.02, *p*<0.0005; interaction Ecotypes x Variants x Conditions F_(2; 36)_=0.29, *p*=0.75. **(B)**Ecotypes _(Can, Kn)_ F_(1; 36)_=90.08, *p*<0.000001; Variants _(NT, O, OMel)_ F_(2; 36)_=42.16, *p*<0.000001; Conditions _(C, NaCl)_ F_(1; 36)_=12.01, *p*<0.005; interaction Ecotypes x Variants F_(2; 36)_=28.62, *p*<0.000001; interaction Ecotypes x Conditions F_(1; 36)_=0.79, *p*=0.38; interaction Variants x Conditions F_(2; 36)_=4.23, *p*<0.05; interaction Ecotypes x Variants x Conditions F_(2; 36)_=2.73, *p*=0.08. (**C**) Ecotypes _(Can, Kn)_ F_(1; 36)_=1489, *p*<0.000001; Variants _(NT, O, OMel)_ F_(2; 36)_=81.71, *p*<0.000001; Conditions _(C, NaCl)_ F_(1; 36)_=22.97, *p*<0.00005; interaction Ecotypes x Variants F_(2; 36)_=15.95, *p*<0.00005; interaction Ecotypes x Conditions F_(1; 36)_=5.98, *p*<0.05; interaction Variants x Conditions F_(2; 36)_=4.40, *p*<0.05; interaction Ecotypes x Variants x Conditions F_(2; 36)_=1.58, *p*=0.22. **(D)** Ecotypes _(Can, Kn)_ F_(1; 36)_=187.48, *p*<0.000001; Variants _(NT, O, OMel)_ F_(2; 36)_=24.33, *p*<0.000001; Conditions _(C, NaCl)_ F_(1; 36)_=1.49, *p*=0.23; interaction Ecotypes x Variants F_(2; 36)_=14.42, *p*<0.00005; interaction Ecotypes x Conditions F_(1; 36)_=1.37, *p*=0.23; interaction Variants x Conditions F_(2; 36)_=1.82, *p*=0.17; interaction Ecotypes x Variants x Conditions F_(2; 36)_=0.7, *p*=0.5.

**SOD – ANOVA results:** (**E)** Ecotypes _(Can, Kn)_ F_(1; 96)_=1.67, *p*=0.2; Variants _(NT, O, OMel)_ F_(2; 96)_=121.83, *p*<0.000001; Conditions _(C, NaCl)_ F_(1; 96)_=243.89, *p*<0.000001; interaction Ecotypes x Variants F_(2; 96)_=12.33, *p*<0.00005; interaction Ecotypes x Conditions F_(1; 96)_=3.17, *p*=0.08; interaction Variants x Conditions F_(2; 96)_=13.72, *p*<0.00001; interaction Ecotypes x Variants x Conditions F_(2; 96)_=1.41, *p*=0.25. **(F)** Ecotypes _(Can, Kn)_ F_(1; 84)_=61.25, *p*<0.000001; Variants _(NT, O, OMel)_ F_(2; 84)_=61.15, *p*<0.000001; Conditions _(C, NaCl)_ F_(1; 84)_=25.59, *p*<0.000005; interaction Ecotypes x Variants F_(2; 84)_=20.19, *p*<0.000001; interaction Ecotypes x Conditions F_(1; 84)_=15.24, *p*<0.0005; interaction Variants x Conditions F_(2; 36)_=1.60, *p*=0.23; interaction Ecotypes x Variants x Conditions F_(2; 84)_=9.76, *p*<0.0005. (**G**) Ecotypes _(Can, Kn)_ F_(1; 96)_=3.52, *p*=0.064; Variants _(NT, O, OMel)_ F_(2; 96)_=50.52, *p*<0.000001; Conditions _(C, NaCl)_ F_(1; 96)_=0.02, *p*=0.88; interaction Ecotypes x Variants F_(2; 96)_=11.96, *p*<0.00005; interaction Ecotypes x Conditions F_(1; 96)_=46.25, *p*<0.000001; interaction Variants x Conditions F_(2; 96)_=20.90, *p*<0.000001; interaction Ecotypes x Variants x Conditions F_(2; 96)_=0.54, *p*=0.58. **(H)**Ecotypes _(Can, Kn)_ F_(1; 96)_=1.75, *p*=0.19; Variants _(NT, O, OMel)_ F_(2; 96)_=100.38, *p*<0.000001; Conditions _(C, NaCl)_ F_(1; 96)_=91.79, *p*<0.000001; interaction Ecotypes x Variants F_(2; 96)_=17.79, *p*<0.000001; interaction Ecotypes x Conditions F_(1; 96)_=0 *p*=0.94; interaction Variants x Conditions F_(2; 96)_=14.27, *p*<0.000005; interaction Ecotypes x Variants x Conditions F_(2; 96)_=0.17, *p*=0.85.

**CAT – ANOVA results:** (**I)** Ecotypes _(Can, Kn)_ F_(1; 60)_=20.64, *p*<0.00005; Variants _(NT, O, OMel)_ F_(2; 60)_=7.06, *p*<0.005; Conditions _(C, NaCl)_ F_(1; 60)_=8.08, *p*<0.01; interaction Ecotypes x Variants F_(2; 60)_=5.1, *p*<0.01; interaction Ecotypes x Conditions F_(1; 60)_=0.05, *p*=0.83; interaction Variants x Conditions F_(2; 60)_=0.78, *p*=0.46; interaction Ecotypes x Variants x Conditions F_(2; 60)_=0.40, *p*=0.68. **(J)** Ecotypes _(Can, Kn)_ F_(1; 84)_=18.81, *p*<0.00005; Variants _(NT, O, OMel)_ F_(2; 84)_=30.19, *p*<0.000001; Conditions _(C, NaCl)_ F_(1; 84)_=451.72, *p*<0.000001; interaction Ecotypes x Variants F_(2; 84)_=39.53, *p*<0.000001; interaction Ecotypes x Conditions F_(1; 84)_=0.035, *p*=0.85; interaction Variants x Conditions F_(2; 84)_=36.16, *p*<0.000001; interaction Ecotypes x Variants x Conditions F_(2; 84)_=18.19, *p*<0.000001. (**K**) Ecotypes _(Can, Kn)_ F_(1; 60)_=43.62, *p*<0.000001; Variants _(NT, O, OMel)_ F_(2; 60)_=1.04, *p*=0.36; Conditions _(C, NaCl)_ F_(1; 60)_=11.50, *p*<0.005; interaction Ecotypes x Variants F_(2; 60)_=0.25, *p*=0.78; interaction Ecotypes x Conditions F_(1; 60)_=0.07, *p*=0.79; interaction Variants x Conditions F_(2; 60)_=0.71, *p*=0.50; interaction Ecotypes x Variants x Conditions F_(2; 60)_=0.54, *p*=0.59. **(L)** Ecotypes _(Can, Kn)_ F_(1; 96)_=17.50, *p*<0.000001; Variants _(NT, O, OMel)_ F_(2; 96)_=17.76, *p*<0.000001; Conditions _(C, NaCl)_ F_(1; 96)_=239.64, *p*<0.000001; interaction Ecotypes x Variants F_(2; 96)_=2.62, *p*=0.078; interaction Ecotypes x Conditions F_(1; 96)_=162.45, *p*<0.000001; interaction Variants x Conditions F_(2; 96)_=14.85, *p*<0.000005; interaction Ecotypes x Variants x Conditions F_(2; 96)_=8.10, *p*<0.001.

**POX – ANOVA results:** (**M)** Ecotypes _(Can, Kn)_ F_(1; 69)_=1300.8, *p*<0.000001; Variants _(NT, O, OMel)_ F_(2; 69)_=1.03, *p*=0.36; Conditions _(C, NaCl)_ F_(1; 69)_=115.11, *p*<0.000001; interaction Ecotypes x Variants F_(2; 69)_=10.00, *p*<0.0005; interaction Ecotypes x Conditions F_(1; 69)_=79.33, *p*<0.000001; interaction Variants x Conditions F_(2; 69)_=103.12, *p*<0.000001; interaction Ecotypes x Variants x Conditions F_(2; 69)_=124.85, *p*<0.000001. **(N)** Ecotypes _(Can, Kn)_ F_(1; 86)_=1072.24, *p*<0.000001; Variants _(NT, O, OMel)_ F_(2; 86)_=99.23, *p*<0.000001; Conditions _(C, NaCl)_ F_(1; 86)_=143.23, *p*<0.000001; interaction Ecotypes x Variants F_(2; 86)_=80.35, *p*<0.000001; interaction Ecotypes x Conditions F_(1; 86)_=174.23, *p*<0.000001; interaction Variants x Conditions F_(2; 86)_=208.84, *p*<0.000001; interaction Ecotypes x Variants x Conditions F_(2; 86)_=127.92, *p*<0.000001. (**O**) Ecotypes _(Can, Kn)_ F_(1; 60)_=125.9, *p*<0.000001; Variants _(NT, O, OMel)_ F_(2; 60)_=1.46, *p*=0.24; Conditions _(C, NaCl)_ F_(1; 60)_=43.87, *p*<0.000001; interaction Ecotypes x Variants F_(2; 60)_=1.86, *p*=0.16; interaction Ecotypes x Conditions F_(1; 60)_=11.91, *p*<0.005; interaction Variants x Conditions F_(2; 60)_=2.71, *p*=0.075; interaction Ecotypes x Variants x Conditions F_(2; 60)_=0.94, *p*=0.40. **(P)** Ecotypes _(Can, Kn)_ F_(1; 96)_=76.65, *p*<0.000001; Variants _(NT, O, OMel)_ F_(2; 96)_=28.39, *p*<0.000001; Conditions _(C, NaCl)_ F_(1; 96)_=222.65, *p*<0.000001; interaction Ecotypes x Variants F_(2; 96)_=36.56, *p*<0.000001; interaction Ecotypes x Conditions F_(1; 96)_=108.81, *p*<0.000001; interaction Variants x Conditions F_(2; 96)_=36.56, *p*<0.000001; interaction Ecotypes x Variants x Conditions F_(2; 96)_=26.21, *p*<0.000001.

**APX – ANOVA results:** (**R)** Ecotypes _(Can, Kn)_ F_(1; 66)_=70.41, *p*<0.000001; Variants _(NT, O, OMel)_ F_(2; 66)_=11.40, *p*<0.0001; Conditions _(C, NaCl)_ F_(1; 66)_=6.11, *p*<0.05; interaction Ecotypes x Variants F_(2; 66)_=3.66, *p*<0.05; interaction Ecotypes x Conditions F_(1; 66)_=7.57, *p*<0.01; interaction Variants x Conditions F_(2; 66)_=0.71, *p*=0.5; interaction Ecotypes x Variants x Conditions F_(2; 66)_=2.30, *p*=0.11. **(S)** Ecotypes _(Can, Kn)_ F_(1; 84)_=67.46, *p*<0.000001; Variants _(NT, O, OMel)_ F_(2; 84)_=129.07, *p*<0.000001; Conditions _(C, NaCl)_ F_(1; 84)_=74.62, *p*<0.000001; interaction Ecotypes x Variants F_(2; 84)_=7.01, *p*<0.005; interaction Ecotypes x Conditions F_(1; 84)_=1.50, *p*=0.22; interaction Variants x Conditions F_(2; 84)_=83.26, *p*<0.000001; interaction Ecotypes x Variants x Conditions F_(2; 84)_=4.69, *p*<0.05. (**T**) Ecotypes _(Can, Kn)_ F_(1; 60)_=13.50, *p*<0.001; Variants _(NT, O, OMel)_ F_(2; 60)_=7.24, *p*<0.005; Conditions _(C, NaCl)_ F_(1; 60)_=12.11, *p*<0.001; interaction Ecotypes x Variants F_(2; 60)_=14.47, *p*<0.00001; interaction Ecotypes x Conditions F_(1; 60)_=0.35, *p*=0.56; interaction Variants x Conditions F_(2; 60)_=1.69, *p*=0.19; interaction Ecotypes x Variants x Conditions F_(2; 60)_=0.57, *p*=0.57. **(U)** Ecotypes _(Can, Kn)_ F_(1; 95)_=26.12, *p*<0.000001; Variants _(NT, O, OMel)_ F_(2; 95)_=34.87, *p*<0.000001; Conditions _(C, NaCl)_ F_(1; 95)_=27.68, *p*<0.000005; interaction Ecotypes x Variants F_(2; 95)_=2.42, *p*=0.09; interaction Ecotypes x Conditions F_(1; 95)_=3.45, *p*=0.066; interaction Variants x Conditions F_(2; 95)_=30.56, *p*<0.000001; interaction Ecotypes x Variants x Conditions F_(2; 95)_=0.306, *p*=0.74.

**Fig. 6: Ethylene – ANOVA results:** Ecotypes _(Can, Kn)_ F_(1; 27)_=0,53, *p*=0.47; Variants _(NT, O, OMel)_ F_(2; 27)_=19.50, *p*<0.00001; Conditions _(C, NaCl)_ F_(1; 27)_=67.23, *p*<0.000001; interaction Ecotypes x Variants F_(2; 27)_=6.57, *p*<0.005; interaction Ecotypes x Conditions F_(1; 27)_=10.27, *p*<0.005; interaction Variants x Conditions F_(2; 27)_=0.77, *p*=0.47; interaction Ecotypes x Variants x Conditions F_(2; 27)_=1.93, *p*=0.16.

**Supplementary figures**

**Fig. S2. Akita:** Variants _(NT, O, OMel)_ F_(2; 12)_=43.78, *p*<0.000005; Conditions _(C, NaCl)_ F_(1; 12)_=51.6, *p*<0.00005; interaction Variants x Conditions: F_(2; 12)_=0.23, *p*=0.80

**Alc-0:** Variants _(NT, O, OMel)_ F_(2; 12)_=27.79, *p*<0.00005; Conditions _(C, NaCl)_ F_(1; 12)_=14.43, *p*<0.005; interaction Variants x Conditions F_(2; 12)_=2.78, *p*=0.10.

**Amel-1:** Variants _(NT, O, OMel)_ F_(2; 12)_=26.09, *p*<0.00005; Conditions _(C, NaCl)_ F_(1; 12)_=19.11, *p*<0.001; interaction Variants x Conditions F_(2; 12)_=1.76, *p*=0.21.

**Bl-1:** Variants _(NT, O, OMel)_ F_(2; 12)_=10.02, *p*<0.005; Conditions _(C, NaCl)_ F_(1; 12)_=6.35, *p*<0.05; interaction Variants x Conditions F_(2; 12)_=0.37, *p*=0.7.

**Blh-1:** Variants _(NT, O, OMel)_ F_(2; 12)_=11.91, *p*<0.005; Conditions _(C, NaCl)_ F_(1; 12)_=26.72, *p*<0.0005; interaction Variants x Conditions F_(2; 12)_=4.99, *p*<0.05.

**Bur-0:** Variants _(NT, O, OMel)_ F_(2; 12)_=14.08, *p*<0.001; Conditions _(C, NaCl)_ F_(1; 12)_=5.67, *p*<0.05; interaction Variants x Conditions F_(2; 12)_=1.70, *p*=0.22.

**Can-0:** Variants _(NT, O, OMel)_ F_(2; 12)_=5.9, *p*<0.05; Conditions _(C, NaCl)_ F_(1; 12)_=1.42, *p*=0.26; interaction Variants x Conditions F_(2; 12)_=0.14, *p*=0.87.

**Col-0:** Variants _(NT, O, OMel)_ F_(2; 12)_=11.47, *p*<0.005; Conditions _(C, NaCl)_ F_(1; 12)_=6.42, *p*<0.05; interaction Variants x Conditions F_(2; 12)_=1.48, *p*=0.27.

**Ct-1:** Variants _(NT, O, OMel)_ F_(2; 12)_=25.27, *p*<0.00005; Conditions _(C, NaCl)_ F_(1; 12)_=7.18, *p*<0.05; interaction Variants x Conditions F_(2; 12)_=0.33, *p*=0.73.

**Cvi-0:** Variants _(NT, O, OMel)_ F_(2; 12)_=32.99, *p*<0.00005; Conditions _(C, NaCl)_ F_(1; 12)_=6.22, *p*<0.05; interaction Variants x Conditions F_(2; 12)_=0.18, *p*=0.83.

**Dew-2:** Variants _(NT, O, OMel)_ F_(2; 12)_=21.02, *p*<0.0005; Conditions _(C, NaCl)_ F_(1; 12)_=4.69, *p*=0.051; interaction Variants x Conditions F_(2; 12)_=1.31, *p*=0.31.

**Dhs-2:** Variants _(NT, O, OMel)_ F_(2; 12)_=20.95, *p*<0.0005; Conditions _(C, NaCl)_ F_(1; 12)_=33.79, *p*<0.0001; interaction Variants x Conditions F_(2; 12)_=1.85, *p*=0.20.

**Edi-0:** Variants _(NT, O, OMel)_ F_(2; 12)_=20.29, *p*<0.0005; Conditions _(C, NaCl)_ F_(1; 12)_=25.15, *p*<0.0005; interaction Variants x Conditions F_(2; 12)_=4.55, *p*<0.05.

**Ge-0:** Variants _(NT, O, OMel)_ F_(2; 12)_=4.87, *p*<0.05; Conditions _(C, NaCl)_ F_(1; 12)_=5.46, *p*<0.05; interaction Variants x Conditions F_(2; 12)_=0.38, *p*=0.69.

**Gre-0:** Variants _(NT, O, OMel)_ F_(2; 12)_=84.41, *p*<0.0000001; Conditions _(C, NaCl)_ F_(1; 12)_=11.14, *p*<0.01; interaction Variants x Conditions F_(2; 12)_=0.71, *p*=0.51.

**Jea:** Variants _(NT, O, OMel)_ F_(2; 12)_=43.02, *p*<0.000005; Conditions _(C, NaCl)_ F_(1; 12)_=1.42, *p*=0.26; interaction Variants x Conditions F_(2; 12)_=0.13, *p*=0.88.

**Kn-0 (n=9-14):** Variants _(NT, O, OMel)_ F_(2; 12)_=68.52, *p*<0.0000005; Conditions _(C, NaCl)_ F_(1; 12)_=19.14, *p*<0.001; interaction Variants x Conditions F_(2; 12)_=3.34, *p*=0.07.

**Less-1:** Variants _(NT, O, OMel)_ F_(2; 12)_=19.96, *p*<0.0005; Conditions _(C, NaCl)_ F_(1; 12)_=3.30, *p*=0.09; interaction Variants x Conditions F_(2; 12)_=0.21, *p*=0.82.

**LI-0:** Variants _(NT, O, OMel)_ F_(2; 12)_=53.04, *p*<0.000005; Conditions _(C, NaCl)_ F_(1; 12)_=25.29, *p*<0.0005; interaction Variants x Conditions F_(2; 12)_=1.78, *p*=0.21.

**Mh-1:** Variants _(NT, O, OMel)_ F_(2; 12)_=55.57, *p*<0.000001; Conditions _(C, NaCl)_ F_(1; 12)_=21.35, *p*<0.001; interaction Variants x Conditions F_(2; 12)_=3.14, *p*=0.08.

**Mt-0:** Variants _(NT, O, OMel)_ F_(2; 12)_=49.71, *p*<0.000005; Conditions _(C, NaCl)_ F_(1; 12)_=10.33, *p*<0.01; interaction Variants x Conditions F_(2; 12)_=1.06, *p*=0.38.

**N13:** Variants _(NT, O, OMel)_ F_(2; 12)_=23.24, *p*<0.0001; Conditions _(C, NaCl)_ F_(1; 12)_=14.41, *p*<0.005; interaction Variants x Conditions F_(2; 12)_=1.61, *p*=0.24.

**Nok-1:** Variants _(NT, O, OMel)_ F_(2; 12)_=36.91, *p*<0.00001; Conditions _(C, NaCl)_ F_(1; 12)_=10.43, *p*<0.01; interaction Variants x Conditions F_(2; 12)_=2.79, *p*=0.10.

**Oy-0:** Variants _(NT, O, OMel)_ F_(2; 12)_=45.88, *p*<0.000005; Conditions _(C, NaCl)_ F_(1; 12)_=15.57, *p*<0.005; interaction Variants x Conditions F_(2; 12)_=0.01, *p*=0.99.

**Pyl-1:** Variants _(NT, O, OMel)_ F_(2; 12)_=9.42, *p*<0.005; Conditions _(C, NaCl)_ F_(1; 12)_=7.95, *p*<0.05; interaction Variants x Conditions F_(2; 12)_=1.96, *p*=0.18.

**Sakata:** Variants _(NT, O, OMel)_ F_(2; 12)_=0.60, *p*=0.56; Conditions _(C, NaCl)_ F_(1; 12)_=39.93, *p*<0.00005; interaction Variants x Conditions F_(2; 12)_=0.25, *p*=0.79.

**Sha:** Variants _(NT, O, OMel)_ F_(2; 12)_=27.42, *p*<0.00005; Conditions _(C, NaCl)_ F_(1; 12)_=14.07, *p*<0.005; interaction Variants x Conditions F_(2; 12)_=1.62, *p*=0.24.

**St-0:** Variants _(NT, O, OMel)_ F_(2; 12)_=10.38, *p*<0.005; Conditions _(C, NaCl)_ F_(1; 12)_=9.14, *p*<0.05; interaction Variants x Conditions F_(2; 12)_=1.97, *p*=0.18.

**Stw-0:** Variants _(NT, O, OMel)_ F_(2; 12)_=69.55, *p*<0.0000005; Conditions _(C, NaCl)_ F_(1; 12)_=14.66, *p*<0.005; interaction Variants x Conditions F_(2; 12)_=0.75, *p*=0.49.

**Tsu-0:** Variants _(NT, O, OMel)_ F_(2; 12)_=92.03, *p*<0.0000001; Conditions _(C, NaCl)_ F_(1; 12)_=1.65, *p*=0.22; interaction Variants x Conditions F_(2; 12)_=0.17, *p*=0.85.

**Fig. S3. Akita:** Variants _(NT, O, OMel)_ F_(2; 80)_=20.03, *p*<0.0000001; Conditions _(C, NaCl)_ F_(1; 80)_=197.23, *p*<0.0000001; interaction Variants x Conditions: F_(2; 80)_=5.91, *p*<0. 005

**Alc-0:** Variants _(NT, O, OMel)_ F_(2; 72)_=53.56, *p*<0.0000001; Conditions _(C, NaCl)_ F_(1; 72)_=32.90, *p*<0.0000001; interaction Variants x Conditions F_(2; 72)_=3.73, *p*<0.05.

**Amel-1:** Variants _(NT, O, OMel)_ F_(2; 76)_=19.76, *p*<0.0000001; Conditions _(C, NaCl)_ F_(1; 76)_=87.70, *p*<0.0000001; interaction Variants x Conditions F_(2; 76)_=1.47, *p*=0.237.

**Bl-1:** Variants _(NT, O, OMel)_ F_(2; 73)_=70.55, *p*<0.0000001; Conditions _(C, NaCl)_ F_(1; 73)_=14.96, *p*<0.0005; interaction Variants x Conditions F_(2; 73)_=10.82, *p*<0.0001.

**Blh-1:** Variants _(NT, O, OMel)_ F_(2; 52)_=1.189, *p*=0.313; Conditions _(C, NaCl)_ F_(1; 52)_=19.63, *p*<0.00005; interaction Variants x Conditions F_(2; 52)_=10.49, *p*<0.0005.

**Bur-0:** Variants _(NT, O, OMel)_ F_(2; 61)_=5.95, *p*<0.005; Conditions _(C, NaCl)_ F_(1; 61)_=397.60, *p*<0.0000001; interaction Variants x Conditions F_(2; 61)_=38.47, *p*<0.0000001.

**Can-0:** Variants _(NT, O, OMel)_ F_(2; 63)_=15.93, *p*<0.000005; Conditions _(C, NaCl)_ F_(1; 63)_=254.63, *p*<0.0000001; interaction Variants x Conditions F_(2; 63)_=4.46, *p*<0.05.

**Col-0:** Variants _(NT, O, OMel)_ F_(2; 65)_=7.65, *p*<0.0010; Conditions _(C, NaCl)_ F_(1; 65)_=312.4, *p*<0.0000001; interaction Variants x Conditions F_(2; 65)_=0.67, *p*=0.514.

**Ct-1:** Variants _(NT, O, OMel)_ F_(2; 75)_=28.25, *p*<0.0000001; Conditions _(C, NaCl)_ F_(1; 75)_=301.68, *p*<0.0000001; interaction Variants x Conditions F_(2; 75)_=2.76, *p*=0.0697.

**Cvi-0:** Variants _(NT, O, OMel)_ F_(2; 57)_=49.55, *p*<0.0000001; Conditions _(C, NaCl)_ F_(1; 57)_=3.042, *p*=0.0865; interaction Variants x Conditions F_(2; 57)_=10.571, *p*<0.0005.

**Dew-2:** Variants _(NT, O, OMel)_ F_(2; 52)_=7.61, *p*<0.005; Conditions _(C, NaCl)_ F_(1; 52)_=3.24, *p*=0.0774; interaction Variants x Conditions F_(2; 52)_=5.27, *p*<0.01.

**Dhs-2:** Variants _(NT, O, OMel)_ F_(2; 63)_=13.32, *p*<0.00005; Conditions _(C, NaCl)_ F_(1; 63)_=61.23, *p*<0.0000001; interaction Variants x Conditions F_(2; 63)_=1.09, *p*=0.341.

**Edi-0:** Variants _(NT, O, OMel)_ F_(2; 66)_=39.25, *p*<0.0000001; Conditions _(C, NaCl)_ F_(1; 66)_=69.9, *p*<0.0000001; interaction Variants x Conditions F_(2; 66)_=1.44, *p*=0.243.

**Ge-0:** Variants _(NT, O, OMel)_ F_(2; 64)_=11.08, *p*<0.0001; Conditions _(C, NaCl)_ F_(1; 64)_=55.07, *p*<0.0000001; interaction Variants x Conditions F_(2; 64)_=3.83, *p*<0.05.

**Gre-0:** Variants _(NT, O, OMel)_ F_(2; 72)_=26.85, *p*<0.0000001; Conditions _(C, NaCl)_ F_(1; 72)_=315.80, *p*<0.0000001; interaction Variants x Conditions F_(2; 72)_=8.35, *p*<0.001.

**Jea:** Variants _(NT, O, OMel)_ F_(2; 66)_=24.5, *p*<0.0000001; Conditions _(C, NaCl)_ F_(1; 66)_=252.06, *p*<0.000001; interaction Variants x Conditions F_(2; 66)_=1.53, *p*=0.224.

**Kn-0:** Variants _(NT, O, OMel)_ F_(2; 68)_=42.31, *p*<0.0000001; Conditions _(C, NaCl)_ F_(1; 68)_=175.08, *p*<0.000001; interaction Variants x Conditions F_(2; 68)_=7.39, *p*<0.05.

**Less-1:** Variants _(NT, O, OMel)_ F_(2; 64)_=38.27, *p*<0.0000001; Conditions _(C, NaCl)_ F_(1; 64)_=80.66, *p*<0.0000001; interaction Variants x Conditions F_(2; 64)_=0.45, *p*=0.617.

**LI-0:** Variants _(NT, O, OMel)_ F_(2; 55)_=42.46, *p*<0.0000001; Conditions _(C, NaCl)_ F_(1; 55)_=137.45, *p*<0.0000001; interaction Variants x Conditions F_(2; 55)_=3.19, *p*<0.05.

**Mh-1:** Variants _(NT, O, OMel)_ F_(2; 62)_=63.49, *p*<0.0000001; Conditions _(C, NaCl)_ F_(1; 62)_=140.07, *p*<0.0000001; interaction Variants x Conditions F_(2; 62)_=0.67, *p*=0.517.

**Mt-0:** Variants _(NT, O, OMel)_ F_(2; 65)_=27.30, *p*<0.0000001; Conditions _(C, NaCl)_ F_(1; 65)_=195.69, *p*<0.0000001; interaction Variants x Conditions F_(2; 65)_=1.37, *p*=0.262.

**N13:** Variants _(NT, O, OMel)_ F_(2; 62)_=0.499, *p*=0.609; Conditions _(C, NaCl)_ F_(1; 62)_=115.99, *p*<0.0000001; interaction Variants x Conditions F_(2; 62)_=2.36, *p*=0.103.

**Nok-1:** Variants _(NT, O, OMel)_ F_(2; 65)_=2.62, *p*=0.08; Conditions _(C, NaCl)_ F_(1; 65)_=41.39, *p*<0.0000001; interaction Variants x Conditions F_(2; 65)_=1.56, *p*=0.218.

**Oy-0:** Variants _(NT, O, OMel)_ F_(2; 62)_=44.86, *p*<0.0000001; Conditions _(C, NaCl)_ F_(1; 62)_=277.36, *p*<0.0000001; interaction Variants x Conditions F_(2; 62)_=0.83, *p*=0.44.

**Pyl-1:** Variants _(NT, O, OMel)_ F_(2; 63)_=62.09, *p*<0.0000001; Conditions _(C, NaCl)_ F_(1; 63)_=352.83, *p*<0.0000001; interaction Variants x Conditions F_(2; 63)_=47.86, *p*<0.0000001.

**Sakata:** Variants _(NT, O, OMel)_ F_(2; 59)_=80.04, *p*<0.0000001; Conditions _(C, NaCl)_ F_(1; 59)_=636.83, *p*<0.0000001; interaction Variants x Conditions F_(2; 59)_=18.98, *p*<0.0000001.

**Sha:** Variants _(NT, O, OMel)_ F_(2; 72)_=11.59, *p*<0.00005; Conditions _(C, NaCl)_ F_(1; 72)_=347.56, *p*<0.0000001; interaction Variants x Conditions F_(2; 72)_=1.26, *p*=0.29

**St-0:** Variants _(NT, O, OMel)_ F_(2; 55)_=13.91, *p*<0.00005; Conditions _(C, NaCl)_ F_(1; 55)_=500.89, *p*<0.0000001; interaction Variants x Conditions F_(2; 55)_=3.05, *p*=0.0556.

**Stw-0:** Variants _(NT, O, OMel)_ F_(2; 73)_=21.62, *p*<0.0000001; Conditions _(C, NaCl)_ F_(1; 73)_=99.52, *p*<0.0000001; interaction Variants x Conditions F_(2; 73)_=10.53, *p*<0.0001.

**Tsu-0:** Variants _(NT, O, OMel)_ F_(2; 57)_=16.76, *p*<0.000002; Conditions _(C, NaCl)_ F_(1; 57)_=18.86, *p*<0.0001; interaction Variants x Conditions F_(2; 57)_=1.56, *p*=0.219.

**Fig. S4 Akita:** Variants _(NT, O, OMel)_ F_(2; 80)_=8.22, *p*<0.001; Conditions _(C, NaCl)_ F_(1; 80)_=65.35, *p*<0.0000001; interaction Variants x Conditions F_(2; 80)_=3.48, *p*<0.05.

**Alc-0:** Variants _(NT, O, OMel)_ F_(2; 72)_=44.90, *p*<0.0000001; Conditions _(C, NaCl)_ F_(1; 72)_=26.11, *p*<0.000005; interaction Variants x Conditions F_(2; 72)_=5.57, *p*<0.01.

**Amel-1:** Variants _(NT, O, OMel)_ F_(2; 76)_=26.12, *p*<0.0000001; Conditions _(C, NaCl)_ F_(1; 76)_=99.45, *p*<0.0000001; interaction Variants x Conditions F_(2; 76)_=10.14, *p*<0.0005.

**Bl-1:** Variants _(NT, O, OMel)_ F_(2; 73)_=35.24, *p*<0.0000001; Conditions _(C, NaCl)_ F_(1; 73)_=109.64, *p*<0.0000001; interaction Variants x Conditions F_(2; 73)_=0.028, *p*=0.972.

**Blh-1:** Variants _(NT, O, OMel)_ F_(2; 52)_=6.25, *p*<0.005; Conditions _(C, NaCl)_ F_(1; 52)_=72.29, *p*<0.0000001; interaction Variants x Conditions F_(2; 52)_=6.14, *p*<0.0040.

**Bur-0:** Variants _(NT, O, OMel)_ F_(2; 61)_=2.20, *p*=0.120; Conditions _(C, NaCl)_ F_(1; 61)_=171.08, *p*<0.0000001; interaction Variants x Conditions F_(2; 61)_=5.03, *p*<0.01.

**Can-0:** Variants _(NT, O, OMel)_ F_(2; 63)_=11.17, *p*<0.0001; Conditions _(C, NaCl)_ F_(1; 63)_=47.72, *p*<0.0000001; interaction Variants x Conditions F_(2; 63)_=11.44, *p*<0.0001.

**Col-0:** Variants _(NT, O, OMel)_ F_(2; 65)_=8.48, *p*<0.001; Conditions _(C, NaCl)_ F_(1; 65)_=109.86, *p*<0.0000001; interaction Variants x Conditions F_(2; 65)_=4.78, *p*<0.005.

**Ct-1:** Variants _(NT, O, OMel)_ F_(2; 75)_=19.09, *p*<0.0000001; Conditions _(C, NaCl)_ F_(1; 75)_=52.88, *p*<0.0000001; interaction Variants x Conditions F_(2; 75)_=2.71, *p*=0.0732.

**Cvi-0:** Variants _(NT, O, OMel)_ F_(2; 57)_=52.30, *p*<0.0000001; Conditions _(C, NaCl)_ F_(1; 57)_=27.89, *p*<0.000005; interaction Variants x Conditions F_(2; 57)_=0.162, *p*=0.851.

**Dew-2:** Variants _(NT, O, OMel)_ F_(2; 52)_=23.78, *p*<0.0000001; Conditions _(C, NaCl)_ F_(1; 52)_=16.76, *p*<0.0005; interaction Variants x Conditions F_(2; 52)_=2.94, *p*=0.0615.

**Dhs-2:** Variants _(NT, O, OMel)_ F_(2; 63)_=2.52, *p*=0.088; Conditions _(C, NaCl)_ F_(1; 63)_=11.22, *p*<0.005; interaction Variants x Conditions F_(2; 63)_=0.017, *p*=0.983.

**Edi-0:** Variants _(NT, O, OMel)_ F_(2; 66)_=19.95, *p*<0.0000001; Conditions _(C, NaCl)_ F_(1; 66)_=33.55, *p*<0.0000001; interaction Variants x Conditions F_(2; 66)_=3.92, *p*<0.05.

**Ge-0:** Variants _(NT, O, OMel)_ F_(2; 64)_=8.39, *p*<0.001; Conditions _(C, NaCl)_ F_(1; 64)_=67.49, *p*<0.0000001; interaction Variants x Conditions F_(2; 64)_=3.92, *p*<0.05.

**Gre-0:** Variants _(NT, O, OMel)_ F_(2; 72)_=60.55, *p*<0.0000001; Conditions _(C, NaCl)_ F_(1; 72)_=112.90, *p*<0.0000001; interaction Variants x Conditions F_(2; 72)_=8.69, *p*<0.0005.

**Jea:** Variants _(NT, O, OMel)_ F_(2; 66)_=55.86, *p*<0.0000001; Conditions _(C, NaCl)_ F_(1; 66)_=105.07, *p*<0.0000001; interaction Variants x Conditions F_(2; 66)_=1.94, *p*=0.152.

**Kn-0:** Variants _(NT, O, OMel)_ F_(2; 68)_=28.13, *p*<0.0000001; Conditions _(C, NaCl)_ F_(1; 68)_=185.43, *p*<0.0000001;; interaction Variants x Conditions F_(2; 68)_=9.66, *p*<0.0005.

**Less-1:** Variants _(NT, O, OMel)_ F_(2; 64)_=26.59, *p*<0.0000001; Conditions _(C, NaCl)_ F_(1; 64)_=111.02, *p*<0.0000001; interaction Variants x Conditions F_(2; 64)_=2.79, *p*=0.069.

**LI-0:** Variants _(NT, O, OMel)_ F_(2; 55)_=32.27, *p*<0.0000001; Conditions _(C, NaCl)_ F_(1; 55)_=48.60, *p*<0.0000001; interaction Variants x Conditions F_(2; 55)_=2.91, *p*=0.0629.

**Mh-1:** Variants _(NT, O, OMel)_ F_(2; 62)_=28.05, *p*<0.0000001; Conditions _(C, NaCl)_ F_(1; 62)_=197.37, *p*<0.0000001; interaction Variants x Conditions F_(2; 62)_=7.07, *p*<0.005.

**Mt-0:** Variants _(NT, O, OMel)_ F_(2; 65)_=67.64, *p*<0.0000001; Conditions _(C, NaCl)_ F_(1; 65)_=73.48, *p*<0.0000001; interaction Variants x Conditions F_(2; 65)_=1.07, *p*=0.350.

**N13:** Variants _(NT, O, OMel)_ F_(2; 62)_=8.39, *p*<0.000595; Conditions _(C, NaCl)_ F_(1; 62)_=78.71, *p*<0.0000001; interaction Variants x Conditions F_(2; 62)_=1.08, *p*=0.345.

**Nok-1:** Variants _(NT, O, OMel)_ F_(2; 65)_=8.53, *p*<0.001; Conditions _(C, NaCl)_ F_(1; 65)_=124.83, *p*<0.0000001; interaction Variants x Conditions F_(2; 65)_=4.74, *p*<0.05.

**Oy-0:** Variants _(NT, O, OMel)_ F_(2; 62)_=60.43, *p*<0.0000001; Conditions _(C, NaCl)_ F_(1; 62)_=44.41, *p*<0.0000001; interaction Variants x Conditions F_(2; 62)_=0.348, *p*=0.707.

**Pyl-1:** Variants _(NT, O, OMel)_ F_(2; 63)_=13.15, *p*<0.00005; Conditions _(C, NaCl)_ F_(1; 63)_=20.44, *p*<0.00005; interaction Variants x Conditions F_(2; 63)_=11.89, *p*<0.00005.

**Sakata:** Variants _(NT, O, OMel)_ F_(2; 59)_=26.16, *p*<0.0000001; Conditions _(C, NaCl)_ F_(1; 59)_=102.71, *p*<0.0000001; interaction Variants x Conditions F_(2; 59)_=12.92, *p*<0.00005.

**Sha:** Variants _(NT, O, OMel)_ F_(2; 72)_=11.23, *p*<0.0001; Conditions _(C, NaCl)_ F_(1; 72)_=22.27, *p*<0.00005; interaction Variants x Conditions F_(2; 72)_=2.12, *p*=0.127.

**St-0:** Variants _(NT, O, OMel)_ F_(2; 55)_=62.97, *p*<0.0000001; Conditions _(C, NaCl)_ F_(1; 55)_=47.81, *p*<0.0000001; interaction Variants x Conditions F_(2; 55)_=6.50, *p*<0.005.

**Stw-0:** Variants _(NT, O, OMel)_ F_(2; 73)_=42.97, *p*<0.0000001; Conditions _(C, NaCl)_ F_(1; 73)_=22.33, *p*<0.00005; interaction Variants x Conditions F_(2; 73)_=8.39, *p*<0.001.

**Tsu-0:** Variants _(NT, O, OMel)_ F_(2; 57)_=46.25, *p*<0.0000001; Conditions _(C, NaCl)_ F_(1; 57)_=48.71, *p*<0.0000001; interaction Variants x Conditions F_(2; 57)_=1.75, *p*=0.184.

**Fig. S5: Akita:** Variants _(NT, O, OMel)_ F_(2; 80)_=63.25, *p*<0.0000001; Conditions _(C, NaCl)_ F_(1; 80)_=447.54, *p*<0.0000001; interaction Variants x Conditions F_(2; 80)_=4.89, *p*<0.01.

**Alc-0:** Variants _(NT, O, OMel)_ F_(2; 72)_=42.76, *p*<0.0000001; Conditions _(C, NaCl)_ F_(1; 72)_=152.81, *p*<0.0000001; interaction Variants x Conditions F_(2; 72)_=36.47, *p*<0.0000001.

**Amel-1:** Variants _(NT, O, OMel)_ F_(2; 76)_=29.91, *p*<0.0000001; Conditions _(C, NaCl)_ F_(1; 76)_=55.04, *p*<0.0000001; interaction Variants x Conditions F_(2; 76)_=29.91, *p*<0.0000001.

**Bl-1:** Variants _(NT, O, OMel)_ F_(2; 73)_=34.65, *p*<0.0000001; Conditions _(C, NaCl)_ F_(1; 73)_=209.96, *p*<0.0000001; interaction Variants x Conditions F_(2; 73)_=3.196, *p*<0.05.

**Blh-1:** Variants _(NT, O, OMel)_ F_(2; 52)_=10.43, *p*<0.0005; Conditions _(C, NaCl)_ F_(1; 52)_=161.28, *p*<0.0000001; interaction Variants x Conditions F_(2; 52)_=15.72, *p*<0.000005.

**Bur-0:** Variants _(NT, O, OMel)_ F_(2; 61)_=45.40, *p*<0.0000001; Conditions _(C, NaCl)_ F_(1; 61)_=324.17, *p*<0.0000001; interaction Variants x Conditions F_(2; 61)_=37.57, *p*<0.0000001.

**Can-0:** Variants _(NT, O, OMel)_ F_(2; 63)_=3.86, *p*<0.05; Conditions _(C, NaCl)_ F_(1; 63)_=106.22, *p*<0.0000001; interaction Variants x Conditions F_(2; 63)_=9.44, *p*<0.0005.

**Col-0:** Variants _(NT, O, OMel)_ F_(2; 65)_=27.91, *p*<0.0000001; Conditions _(C, NaCl)_ F_(1; 65)_=150.55, *p*<0.0000001; interaction Variants x Conditions F_(2; 65)_=7.34, *p*<0.005.

**Ct-1:** Variants _(NT, O, OMel)_ F_(2; 75)_=48.59, *p*<0.0000001; Conditions _(C, NaCl)_ F_(1; 75)_=354.64, *p*<0.0000001; interaction Variants x Conditions F_(2; 75)_=3.90, *p*<0.05.

**Cvi-0:** Variants _(NT, O, OMel)_ F_(2; 57)_=33.79, *p*<0.0000001; Conditions _(C, NaCl)_ F_(1; 57)_=65.09, *p*<0.0000001; interaction Variants x Conditions F_(2; 57)_=9.59, *p*<0.0005.

**Dew-2:** Variants _(NT, O, OMel)_ F_(2; 52)_=70.91, *p*<0.0000001; Conditions _(C, NaCl)_ F_(1; 52)_=59.01, *p*<0.0000001; interaction Variants x Conditions F_(2; 52)_=11.62, *p*<0.0001.

**Dhs-2:** Variants _(NT, O, OMel)_ F_(2; 63)_=4.49, *p*<0.05; Conditions _(C, NaCl)_ F_(1; 63)_=10.38, *p*<0.005; interaction Variants x Conditions F_(2; 63)_=4.49, *p*<0.05.

**Edi-0:** Variants _(NT, O, OMel)_ F_(2; 66)_=16.80, *p*<0.000001; Conditions _(C, NaCl)_ F_(1; 66)_=251.89, *p*<0.0000001; interaction Variants x Conditions F_(2; 66)_=12.04, *p*<0.00005.

**Ge-0:** Variants _(NT, O, OMel)_ F_(2; 64)_=12.10, *p*<0.00005; Conditions _(C, NaCl)_ F_(1; 64)_=165.67, *p*<0.0000001; interaction Variants x Conditions F_(2; 64)_=11.73, *p*<0.00005.

**Gre-0:** Variants _(NT, O, OMel)_ F_(2; 72)_=27.72, *p*<0.0000001; Conditions _(C, NaCl)_ F_(1; 72)_=164.78, *p*<0.0000001; interaction Variants x Conditions F_(2; 72)_=21.15, *p*<0.0000001.

**Jea:** Variants _(NT, O, OMel)_ F_(2; 66)_=88.59, *p*<0.0000001; Conditions _(C, NaCl)_ F_(1; 66)_=283.61, *p*<0.0000001; interaction Variants x Conditions F_(2; 66)_=40.87, *p*<0.0000001.

**Kn-0:** Variants _(NT, O, OMel)_ F_(2; 68)_=116.05; *p*<0.0000001; Conditions _(C, NaCl)_ F_(1; 68)_=408.87; *p*<0.0000001; interaction Variants x Conditions F_(2; 68)_=42.34, *p*<0.0000001.

**Less-1:** Variants _(NT, O, OMel)_ F_(2; 64)_=11.06, *p*<0.0001; Conditions _(C, NaCl)_ F_(1; 64)_=164.97, *p*<0.0000001; interaction Variants x Conditions F_(2; 64)_=6.42, *p*<0.005.

**LI-0:** Variants _(NT, O, OMel)_ F_(2; 55)_=23.26, *p*<0.0000001; Conditions _(C, NaCl)_ F_(1; 55)_=76.44, *p*<0.0000001; interaction Variants x Conditions F_(2; 55)_=11.94, *p*<0.00005.

**Mh-1:** Variants _(NT, O, OMel)_ F_(2; 62)_=32.88, *p*<0.0000001; Conditions _(C, NaCl)_ F_(1; 62)_=156.14, *p*<0.0000001; interaction Variants x Conditions F_(2; 62)_=32.49, *p*<0.0000001.

**Mt-0:** Variants _(NT, O, OMel)_ F_(2; 65)_=117.79, *p*<0.0000001; Conditions _(C, NaCl)_ F_(1; 65)_=230.58, *p*<0.0000001; interaction Variants x Conditions F_(2; 65)_=27.66, *p*<0.0000001.

**N13:** Variants _(NT, O, OMel)_ F_(2; 62)_=16.10, *p*<0.000005; Conditions _(C, NaCl)_ F_(1; 62)_=182.78, *p*<0.0000001; interaction Variants x Conditions F_(2; 62)_=15.40, *p*<0.000005.

**Nok-1:** Variants _(NT, O, OMel)_ F_(2; 65)_=3.19, *p*<0.05; Conditions _(C, NaCl)_ F_(1; 65)_=194.12, *p*<0.0000001; interaction Variants x Conditions F_(2; 65)_=2.92, *p*=0.0610.

**Oy-0:** Variants _(NT, O, OMel)_ F_(2; 62)_=79.88, *p*<0.0000001; Conditions _(C, NaCl)_ F_(1; 62)_=282.19, *p*<0.0000001; interaction Variants x Conditions F_(2; 62)_=40.68, *p*<0.0000001.

**Pyl-1:** Variants _(NT, O, OMel)_ F_(2; 63)_=30.08, *p*<0.0000001; Conditions _(C, NaCl)_ F_(1; 63)_=59.83, *p*<0.0000001; interaction Variants x Conditions F_(2; 63)_=30.08, *p*<0.0000001.

**Sakata:** Variants _(NT, O, OMel)_ F_(2; 59)_=15.01, *p*<0.000005; Conditions _(C, NaCl)_ F_(1; 59)_=164.95, *p*<0.0000001; interaction Variants x Conditions F_(2; 59)_=9.20, *p*<0.0005.

**Sha:** Variants _(NT, O, OMel)_ F_(2; 72)_=16.64, *p*<0.000001; Conditions _(C, NaCl)_ F_(1; 72)_=231.95, *p*<0.0000001; interaction Variants x Conditions F_(2; 72)_=8.24, *p*<0.001.

**St-0:** Variants _(NT, O, OMel)_ F_(2; 55)_=51.65, *p*<0.0000001; Conditions _(C, NaCl)_ F_(1; 55)_=144.61, *p*<0.0000001; interaction Variants x Conditions F_(2; 55)_=26.48, *p*<0.0000001.

**Stw-0:** Variants _(NT, O, OMel)_ F_(2; 73)_=100.36, *p*<0.0000001; Conditions _(C, NaCl)_ F_(1; 73)_=137.05, *p*<0.0000001; interaction Variants x Conditions F_(2; 73)_=11.92, *p*<0.00005.

**Tsu-0:** Variants _(NT, O, OMel)_ F_(2; 57)_=41.56, *p*<0.0000001; Conditions _(C, NaCl)_ F_(1; 57)_=157.80, *p*<0.0000001; interaction Variants x Conditions F_(2; 57)_=18.45, *p*<0.000001.

**Fig. S7: GSH-PX – ANOVA results:** (**A)** Ecotypes _(Can, Kn)_ F_(1; 60)_=6.53, *p*<0.05; Variants _(NT, O, OMel)_ F_(2; 60)_=2.85, *p*=0.066; Conditions _(C, NaCl)_ F_(1; 60)_=0.106, *p*=0.75; interaction Ecotypes x Variants F_(2; 60)_=10.94, *p*<0.0001; interaction Ecotypes x Conditions F_(1; 60)_=0.118, *p*=0.73; interaction Variants x Conditions F_(2; 60)_=0.634, *p*=0.53; interaction Ecotypes x Variants x Conditions F_(2; 60)_=0.992, *p*=0.38. **(B)** Ecotypes _(Can, Kn)_ F_(1; 74)_=36.45, *p*<0.0000001; Variants _(NT, O, OMel)_ F_(2; 74)_=19.23, *p*<0.0000005; Conditions _(C, NaCl)_ F_(1; 74)_=3.69, *p*=0.058; interaction Ecotypes x Variants F_(2; 74)_=11.77, *p*<0.00005; interaction Ecotypes x Conditions F_(1; 74)_=49.51, *p*<0.0000001; interaction Variants x Conditions F_(2; 74)_=58.76, *p*<0.0000001; interaction Ecotypes x Variants x Conditions F_(2; 74)_=0.46, *p*=0.63. (**C**) Ecotypes _(Can, Kn)_ F_(1; 60)_=20.63, *p*<0.00005; Variants _(NT, O, OMel)_ F_(2; 60)_=0.177, *p*=0.84; Conditions _(C, NaCl)_ F_(1; 60)_=10.58, *p*<0.005; interaction Ecotypes x Variants F_(2; 60)_=0.657, *p*=0.52; interaction Ecotypes x Conditions F_(1; 60)_=16.55, *p*>0.0005; interaction Variants x Conditions F_(2; 60)_=0.931, *p*=0.40; interaction Ecotypes x Variants x Conditions F_(2; 60)_=3.68, *p*<0.05. **(D)** Ecotypes _(Can, Kn)_ F_(1; 83)_=169.7, *p*<0.0000001; Variants _(NT, O, OMel)_ F_(2; 83)_=97.38, *p*<0.0000001; Conditions _(C, NaCl)_ F_(1; 83)_=3.48, *p*=0.066; interaction Ecotypes x Variants F_(2; 83)_=0.314, *p*=0.73; interaction Ecotypes x Conditions F_(1; 83)_=42.37, *p*<0.0000001; interaction Variants x Conditions F_(2; 83)_=1.415, *p*=0.25; interaction Ecotypes x Variants x Conditions F_(2; 83)_=17.02, *p*<0.000001.

**GSSG-R – ANOVA results:** (**E)** Ecotypes _(Can, Kn)_ F_(1; 60)_=1.04, *p*=0.31; Variants _(NT, O, OMel)_ F_(2; 60)_=4.51, *p*<0.05; Conditions _(C, NaCl)_ F_(1; 60)_=15.78, *p*<0.0005; interaction Ecotypes x Variants F_(2; 60)_=2.87, *p*=0.065; interaction Ecotypes x Conditions F_(1; 60)_=1.11, *p*=0.30; interaction Variants x Conditions F_(2; 60)_=3.55, *p*<0.05; interaction Ecotypes x Variants x Conditions F_(2; 60)_=6.34, *p*<0.005. **(F)** Ecotypes _(Can, Kn)_ F_(1; 75)_=194.53, *p*<0.000001; Variants _(NT, O, OMel)_ F_(2; 75)_=39.02, *p*<0.000001; Conditions _(C, NaCl)_ F_(1; 75)_=121.56, *p*<0.000001; interaction Ecotypes x Variants F_(2; 75)_=0.56, *p*=0.57; interaction Ecotypes x Conditions F_(1; 75)_=3.67, *p*=0.06; interaction Variants x Conditions F_(2; 75)_=65.03, *p*<0.000001; interaction Ecotypes x Variants x Conditions F_(2; 75)_=31.190, *p*<0.000001. (**G**) Ecotypes _(Can, Kn)_ F_(1; 60)_=30.23, *p*<0.000001; Variants _(NT, O, OMel)_ F_(2; 60)_=0.145, *p*=0.87; Conditions _(C, NaCl)_ F_(1; 60)_=24.79, *p*<0.00001; interaction Ecotypes x Variants F_(2; 60)_=16.80, *p*<0.000005; interaction Ecotypes x Conditions F_(1; 60)_=0.304, *p*=0.58; interaction Variants x Conditions F_(2; 60)_=4.86, *p*<0.05; interaction Ecotypes x Variants x Conditions F_(2; 60)_=0.407, *p*=0.67. **(H)** Ecotypes _(Can, Kn)_ F_(1; 74)_=0.56, *p*=0.46; Variants _(NT, O, OMel)_ F_(2; 74)_=12.45, *p*<0.00005; Conditions _(C, NaCl)_ F_(1; 74)_=26.30, *p*<0.000005; interaction Ecotypes x Variants F_(2; 74)_=7.49, *p*<0.005; interaction Ecotypes x Conditions F_(1; 74)_=19.54, *p*>0.00005; interaction Variants x Conditions F_(2; 74)_=2.93, *p*=0.06; interaction Ecotypes x Variants x Conditions F_(2; 74)_=17.51, *p*<0.000005.

**Fig. S8: Melatonin – ANOVA results:** (**A**) 30 Ecotypes F_(1; 29)_=6.43, *p*<0.00001; (**B**) 30 Ecotypes F_(1; 29)_=19.01, *p*<0.000001; (**C**) 30 Ecotypes F_(1; 29)_=40.42, *p*<0.000001.
