# Supplementary figures and images for: An ecotype-specific effect of osmopriming and melatonin during salt stress in Arabidopsis thaliana
Source: BMC Plant Biol. 2024 Jul 25;24:707. doi: 10.1186/s12870-024-05434-5 (PMC11270801; doi:10.1186/s12870-024-05434-5)

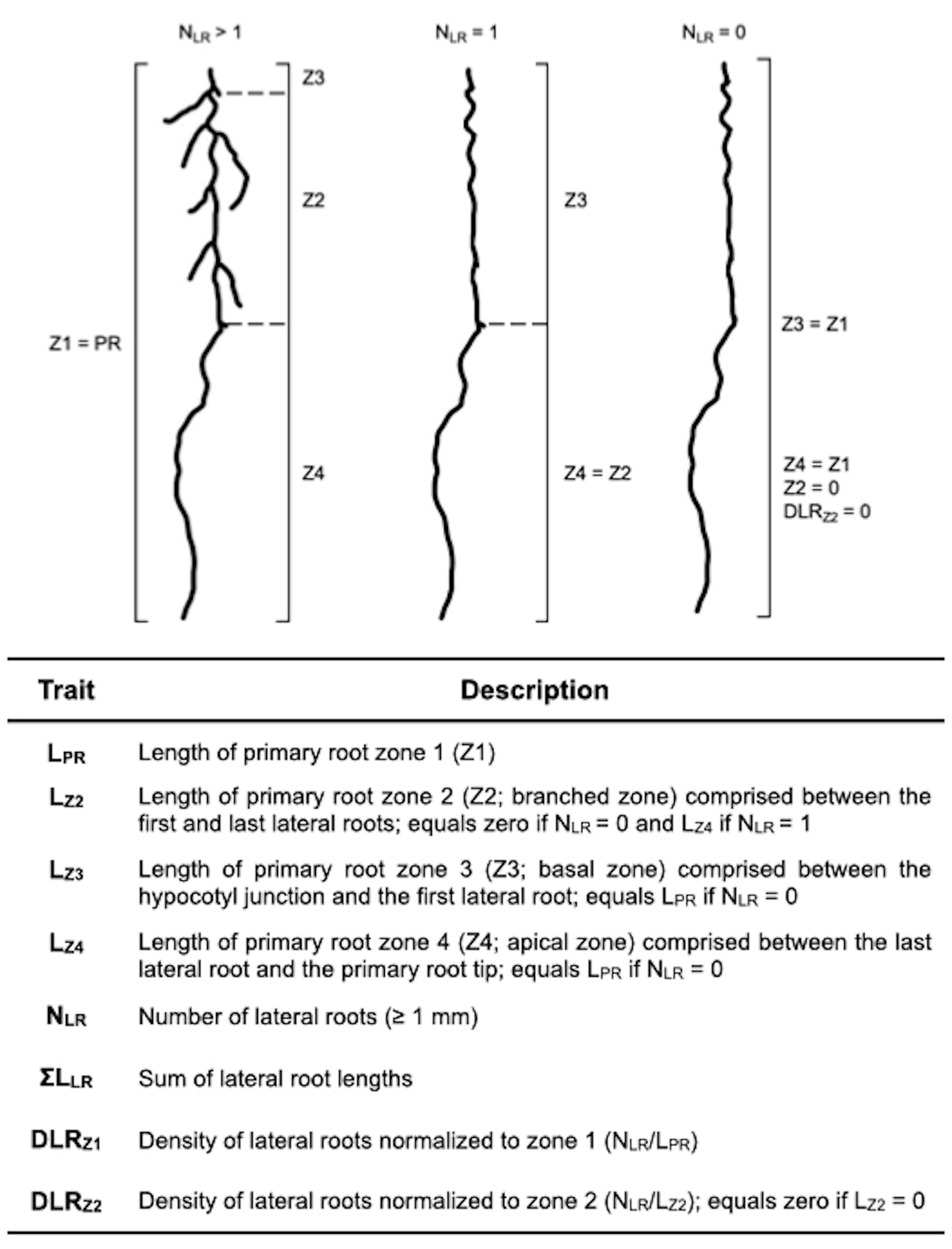

Supplement: Supplementary file 3 — Supplementary Material 3 [file 12870_2024_5434_MOESM3_ESM.jpeg]

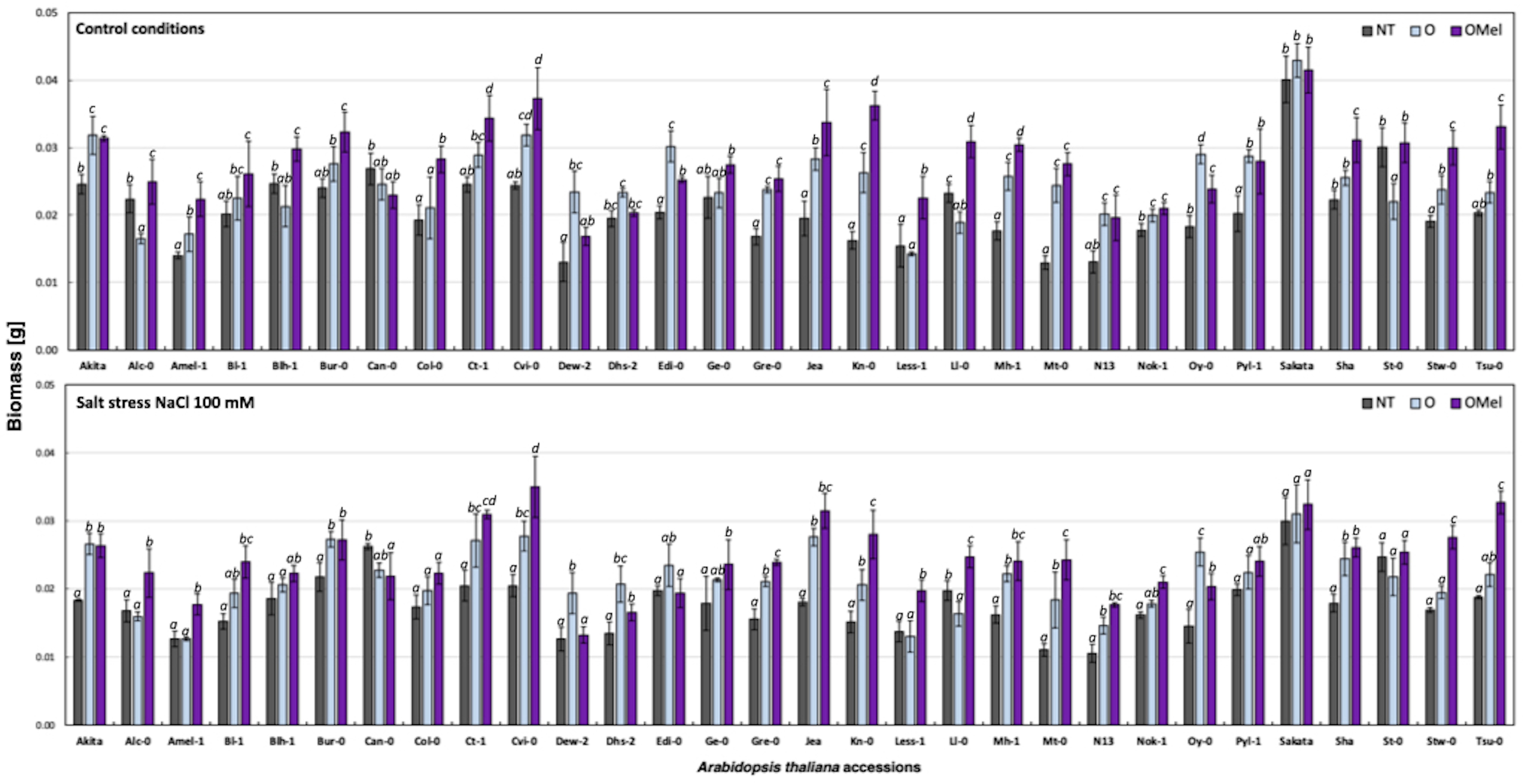

Supplement: Supplementary file 4 — Supplementary Material 4 [file 12870_2024_5434_MOESM4_ESM.jpeg]

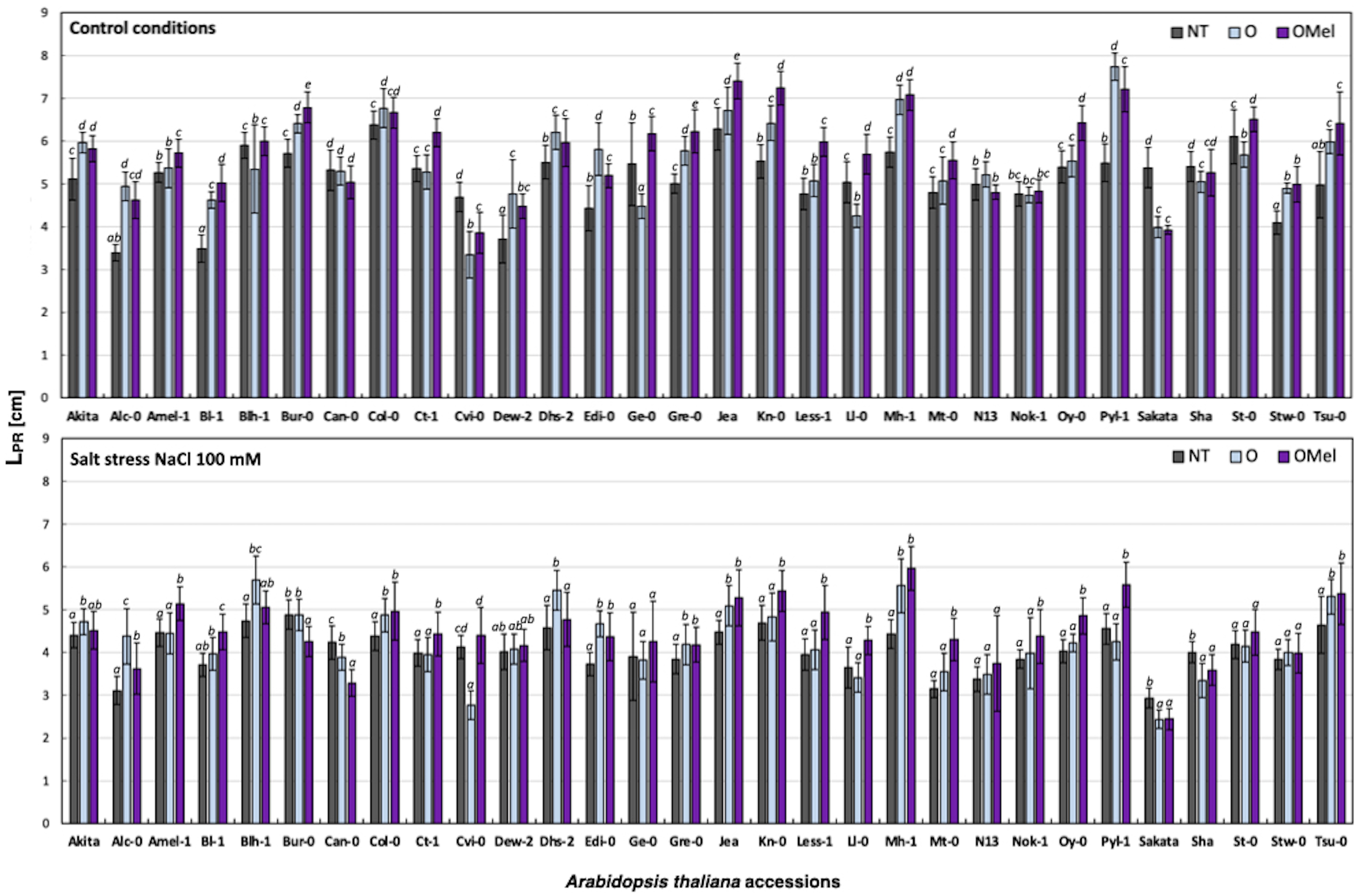

Supplement: Supplementary file 5 — Supplementary Material 5 [file 12870_2024_5434_MOESM5_ESM.jpeg]

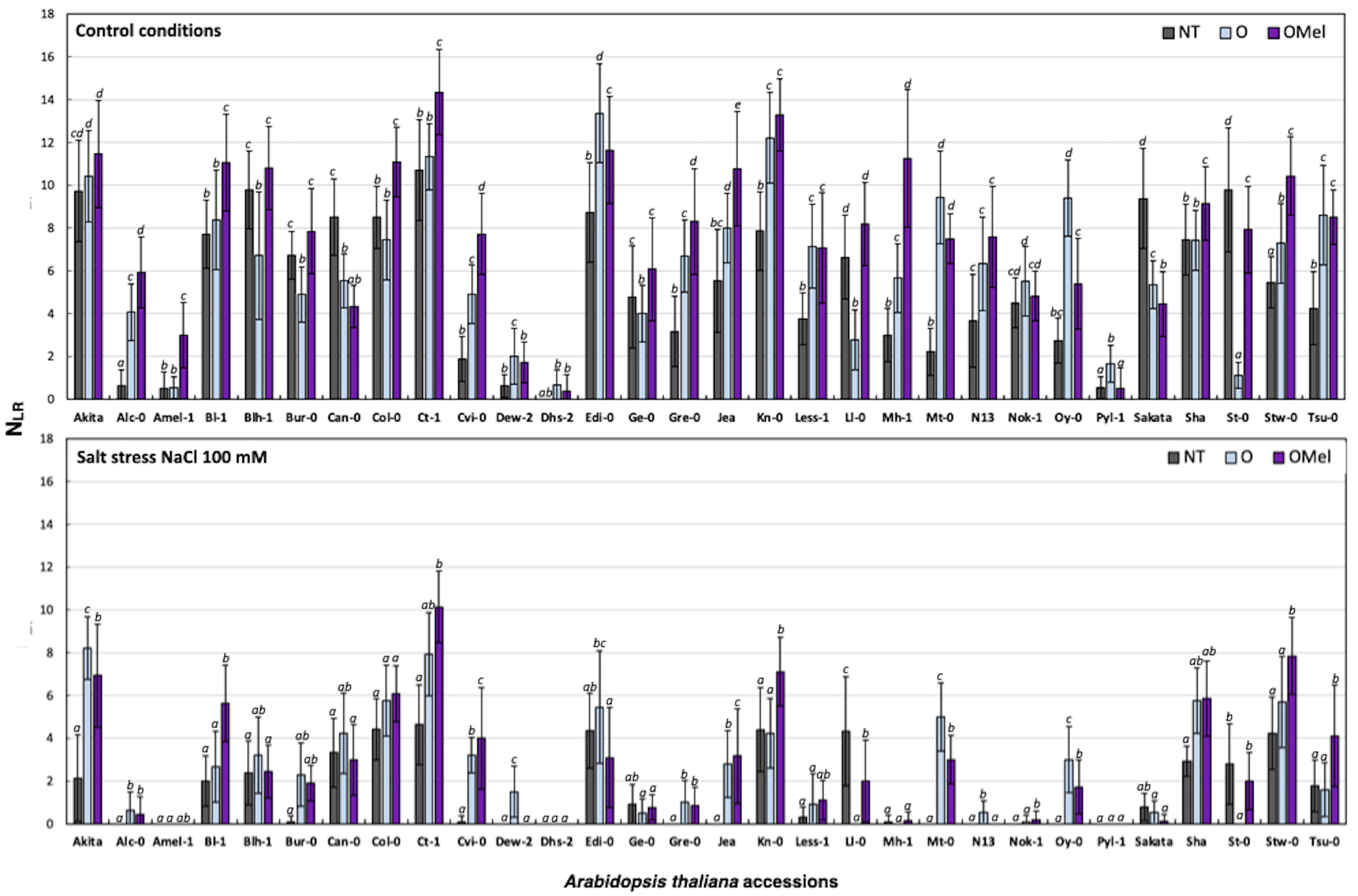

Supplement: Supplementary file 6 — Supplementary Material 6 [file 12870_2024_5434_MOESM6_ESM.jpeg]

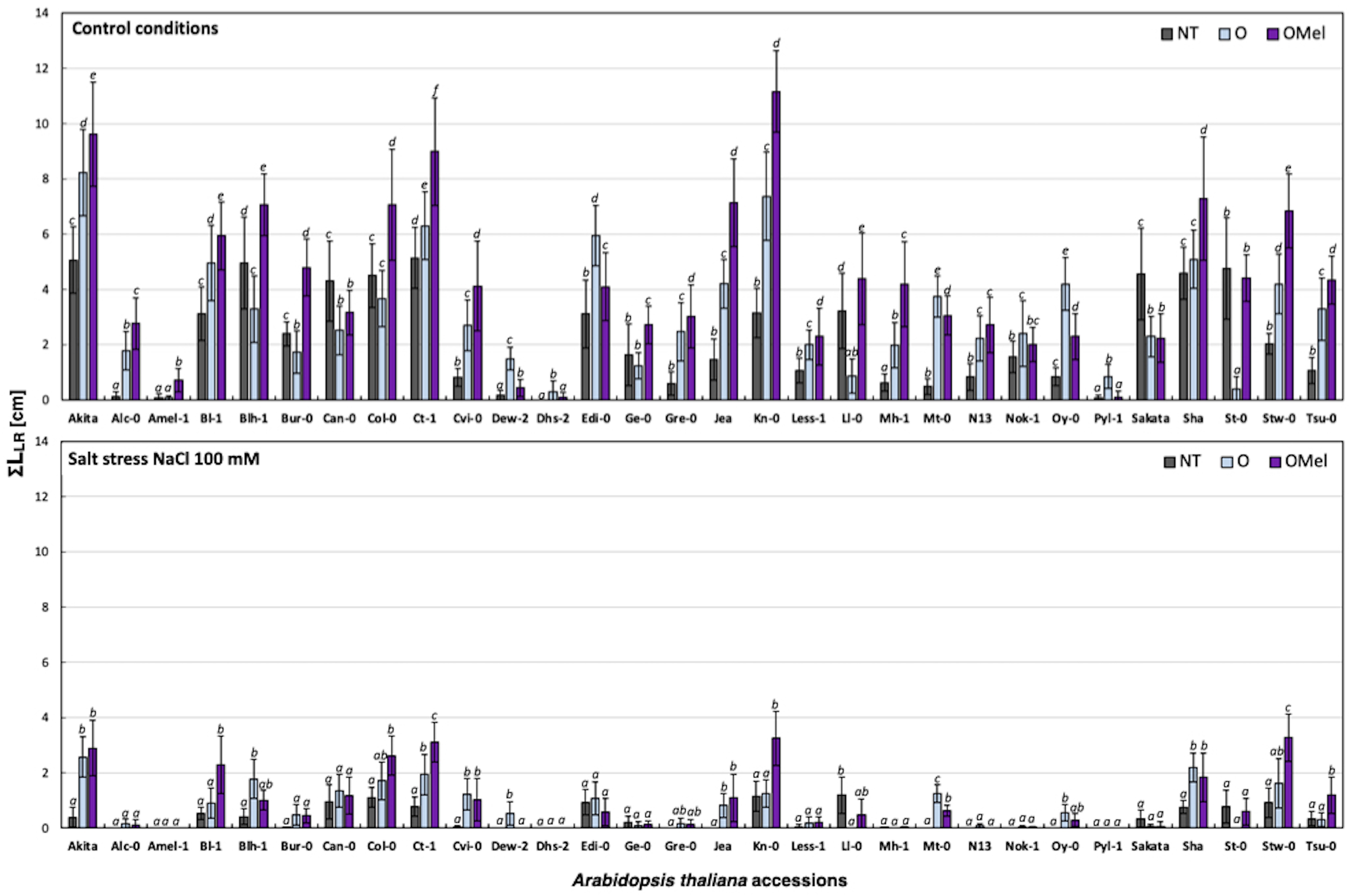

Supplement: Supplementary file 7 — Supplementary Material 7 [file 12870_2024_5434_MOESM7_ESM.jpeg]

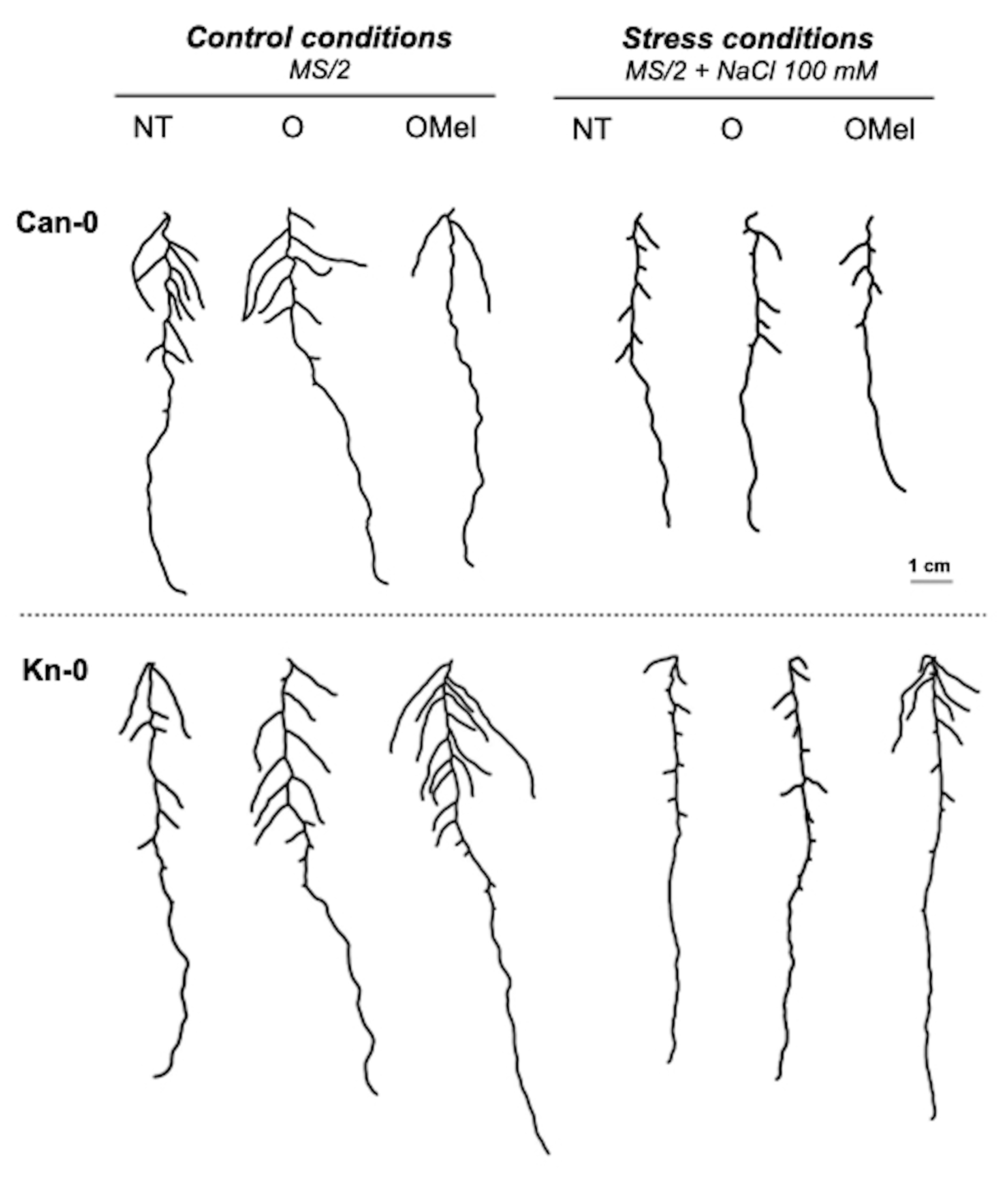

Supplement: Supplementary file 8 — Supplementary Material 8 [file 12870_2024_5434_MOESM8_ESM.jpeg]

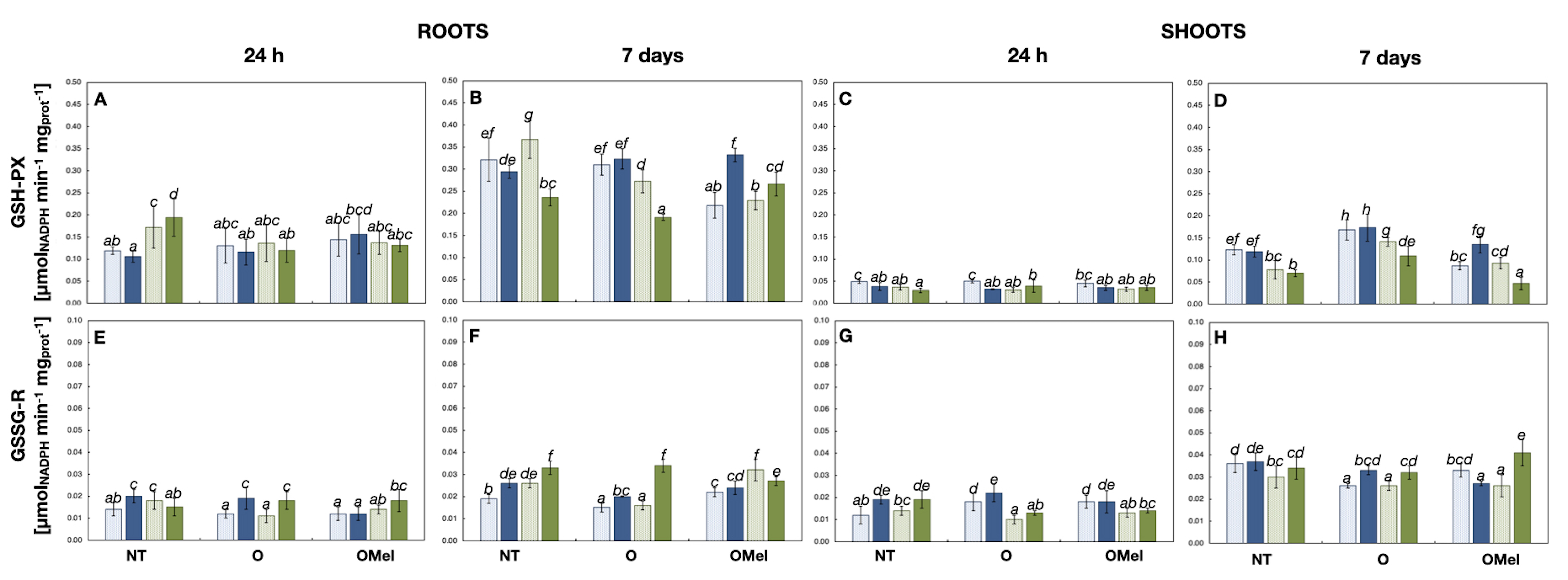

Supplement: Supplementary file 9 — Supplementary Material 9 [file 12870_2024_5434_MOESM9_ESM.jpeg]

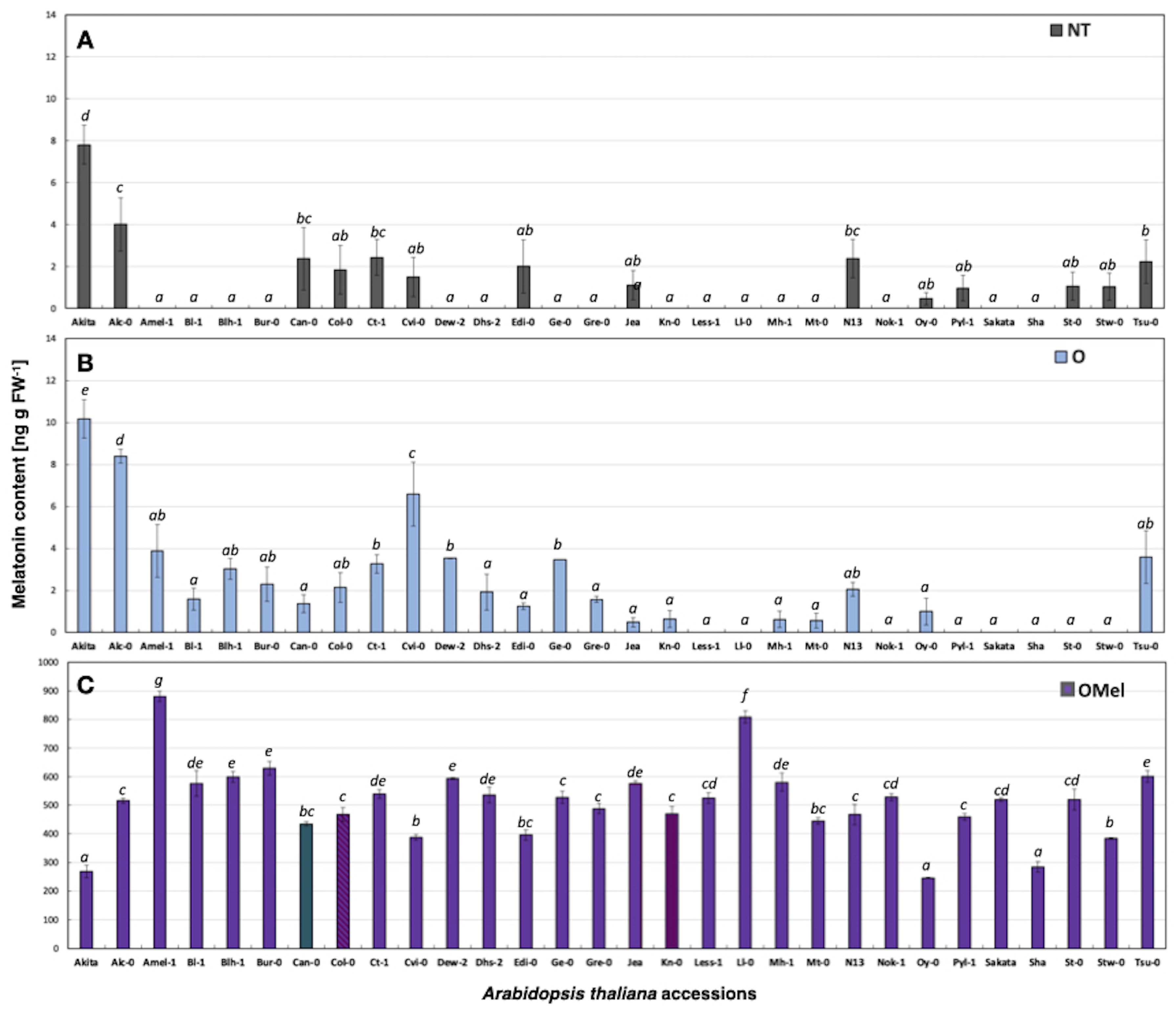

Supplement: Supplementary file 10 — Supplementary Material 10 [file 12870_2024_5434_MOESM10_ESM.jpeg]
